# Comparative Genomics Reveals Metabolic Specificity of *Endozoicomonas* Isolated from a Marine Sponge and the Genomic Repertoire for Host-Bacteria Symbioses

**DOI:** 10.3390/microorganisms7120635

**Published:** 2019-11-30

**Authors:** Anoop Alex, Agostino Antunes

**Affiliations:** 1 CIIMAR/CIMAR, Interdisciplinary Centre of Marine and Environmental Research, University of Porto, 4450-208 Porto, Portugal; 2 Department of Biology, Faculty of Sciences, University of Porto, Rua do Campo Alegre, 4169-007 Porto, Portugal

**Keywords:** *Endozoicomonas*, symbiosis, comparative genomics, sponge–bacteria interaction, eukaryotic-like proteins, secretion systems

## Abstract

The most recently described bacterial members of the genus *Endozoicomonas* have been found in association with a wide variety of marine invertebrates. Despite their ubiquity in the host holobiont, limited information is available on the molecular genomic signatures of the symbiotic association of *Endozoicomonas* with marine sponges. Here, we generated a draft genome of *Endozoicomonas* sp. OPT23 isolated from the intertidal marine sponge *Ophlitaspongia papilla* and performed comprehensive comparative genomics analyses. Genome-specific analysis and metabolic pathway comparison of the members of the genus *Endozoicomonas* revealed the presence of gene clusters encoding for unique metabolic features, such as the utilization of carbon sources through lactate, L-rhamnose metabolism, and a phenylacetic acid degradation pathway in *Endozoicomonas* sp. OPT23. Moreover, the genome harbors genes encoding for eukaryotic-like proteins, such as ankyrin repeats, tetratricopeptide repeats, and Sel1 repeats, which likely facilitate sponge-bacterium attachment. The genome also encodes major secretion systems and homologs of effector molecules that seem to enable the sponge-associated bacterium to interact with the sponge and deliver the virulence factors for successful colonization. In conclusion, the genome analysis of *Endozoicomonas* sp. OPT23 revealed the presence of adaptive genomic signatures that might favor their symbiotic lifestyle within the sponge host.

## 1. Introduction

Sponges (Phylum *Porifera*) interact and co-evolve with microbes belonging to different lineages. Despite the ubiquity of microbes in a wide range of environments, associations between sponge and microbes are not random and often result in sharing of the resources in a particular niche. Moreover, sponge-associated bacteria play a crucial role in sponge biology, metabolism, and ecology [1]. Studies using whole-genome sequencing of microbes isolated from sponges and metagenomic binning approaches have shown the genomic and molecular mechanisms involved in the successful association between the sponges and symbiotic microbes. For instance, genome streamlining [2], evolution of bacterial genome through transposable insertion elements [3], presence of adhesion-related genes, the genes encoding eukaryotic-like protein, effector/virulence factors [4,5,6,7] were reported among several sponge-associated bacteria.

Since the first description of the genus *Endozoicomonas* (Gammaproteobacteria; Oceanospirillales) isolated from the sea slug *Elysia ornate* [8], *Endozoicomonas* has been widely reported to be found in association with different marine invertebrates. For instance, several studies reported the presence of *Endozoicomonas* in sponges, tunicates, cnidarians, annelids, molluscs, and fishes [9,10,11,12,13,14] across large geographical scales (see the review by [15] for further details). Several studies found the predominance of *Endozoicomonas* in healthy Mediterranean gorgonians—*Paramuricea clavata* and coral—*Porites astreoides* when compared to their diseased counterparts [16,17], suggesting that strains belonging to this genus have been considered to be an integral part of the healthy holobiont.

Though numerous studies based on culture-independent analyses using the 16S rRNA (ribosomal RNA) genes have detected the distribution and abundance of *Endozoicomonas*, there are very few reports investigating the physiological capabilities and genomic features of these enigmatic bacterial members of the genus *Endozoicomonas* due to the difficulties in isolation/culturing from the host tissues. So far, only 11 draft genomes of *Endozoicomonas* isolated from sponges, tunicates, coral, mollusc, bryozoa, and fishes are publicly available. Analysis of the draft genomes obtained from bacterial isolates, single cell genomics, and metagenomic binning approaches suggest that *Endozoicomonas* genomes are encoded with a higher frequency of repeat sequences indicating various stages of genome erosion, possible mechanisms involved in host-endosymbiont relationship, enrichment of genes responsible for transporter activity, protein secretion, and transposase, and niche-specific changes through the expansion of virulence genes and loss of metabolic functions [14,18,19].

Despite the abovementioned features detected in the genomes of coral- and fish-associated *Endozoicomonas*, little genomic information is available for sponge-associated *Endozoicomonas*. In this study we sequenced a high-quality draft genome of *Endozoicomonas* sp. OPT23 isolated from the intertidal marine sponge *Ophlitaspongia papilla* and performed a comprehensive comparative genomics analysis with other members (*n* = 11) of the genus to unravel the genomic signatures of the sponge-associated *Endozoicomonas* sp. OPT23.

## 2. Materials and Methods

### 2.1. Isolation of Endozoicomonas

*Endozoicomonas* sp. OPT23 was isolated from the intertidal marine sponge *O. papilla* (Demospongiae) collected from the Atlantic coast of Portugal. Approximately 1 cm^3^ of sponge tissue was washed to remove the loosely associated microbes and other debris, followed by grinding using sterile seawater. The homogenate was serially diluted and spread plated on Difco^TM^ Marine Agar 2216(BD Difco, United Kingdom) medium containing amphotericin B (1 mL/100 mL). Single colonies were obtained after repeated streaking. Bacterial colonies were inoculated into 5 mL tube containing Difco^TM^ Marine Broth 2216 and kept under constant shaking at 28 °C. Genomic DNA was extracted from the bacterial cultures in stationary phase using PureLink^TM^ Genomic DNA kit (Invitrogen, Carlsbad, CA, USA) according to the manufacturer’s protocol for bacterial DNA isolation.

### 2.2. Whole-Genome Sequencing (WGS) and Genome Analyses

WGS of *Endozoicomonas* sp. OPT23 was performed on the Illumina’s HiSeq 2500 Sequencing System using paired-end (PE) read library (2 × 100 nt) with an insert size of ≈350 bp. Prior to assembly, low quality reads (Phred score <30) and adapter sequences were removed using cutadapt v1.12 [20]. The processed reads of >1400-fold coverage (based on a 5 Mb genome size) was assembled using Velvet v1.2.10 [21] with the best possible *k-mer* coverage value (*k* = 99) obtained from VelvetK. Assembly was further improved by scaffolding using SSPACE_Standard v3.0 [22] and gap filling by GapFillerv1.10 [23] followed by the genome annotation using PROKKA v1.12 [24]. Curated dataset constituting bacterial protein sequences from the UniProt Knowledgebase, UniProtKB Release 2017_3 was compiled locally for the functional assignment of predicted coding sequences (CDS). The genome completeness and contamination were determined using CheckM v1.0.08 [25]. Circular view of the sequenced genome was rendered using CGview (Available online: http://wishart.biology.ualberta.ca/cgview/download.html) [26].

The genomes of the members of the genus *Endozoicomonas* (*n* = 11) isolated from different habitats were retrieved from the NCBI (National Center for Biotechnology Information) genomes FTP site [27]. Prior to the comparative genomic analyses all genome datasets were re-annotated using PROKKA v1.12 [24] to avoid the incongruence of different annotation schemes.

### 2.3. Clusters of Orthologous Groups of Proteins

For functional prediction, the protein sequences were queried against locally installed eggNOG database v4.5 [28] using eggnog-mapper tool v1.0.3 (DIAMOND mapping mode and other default settings) [29]. Clusters of orthologous groups (COGs) were retrieved from the eggNOG annotation files using a bash script—eggnog-mapper_COGextraction (Available online: https://github.com/raymondkiu/ eggnog-mapper_COGextraction). A heat map representing the abundance of COG functional class was constructed using heatmap.2 function in gplots package v3.0.1 [30] implemented in R v3.4.4 [31]. Z-test was used to determine the significant differences in proportions of COG categories and considering a *p*-value of <0.01 as threshold for over and under-representation.

### 2.4. Phylogenetic Analyses and Average Nucleotide Identity

The *Endozoicomonas* genome tree was constructed with 622 single-copy orthologous genes identified by OrthoFinder v2.3.1 using default DIAMOND sequence similarity searches [32]. The genes were individually aligned using MUSCLE v3.8.31 [33]. Low quality alignment regions were removed using trimAl v1.4 with ‘automated1′ option [34]. Protein-alignment files were concatenated to a super-alignment (encompassing ≈209,574 sites) using FASconCAT v1.1 [35]. A maximum-likelihood phylogenetic tree was constructed using IQ-TREE v1.6.1 [36] (using 1000 bootstrap replicates) under automated model selection option ‘TEST’. Distantly related bacterial species—*Marinobacterium aestuarii* ST58-10 and *Cobetia marina* JCM 21022—were used as outgroups.

The whole genome-based relatedness among the members of the genus *Endozoicomonas* was calculated using the average nucleotide identity (ANI) and *in silico* DNA-DNA hybridization (DDH) estimates implemented in Pyani [37] and GGDC 2.1 (Genome-to-Genome distance calculator) [38] respectively, with the BLAST algorithm (ANIb). DDH values are estimated with formula 2 due to robustness against the use of incomplete draft genomes. The average nucleotide identity (ANI) values were visualized usingheatmap.2 function in gplots package v3.0.1 [30] implemented in R v3.4.4 [31].

### 2.5. Homolog Clustering and Calculation of Genome-Specific Genes

Protein level homolog clustering, core- and pan-genome sizes of the genus *Endozoicomonas* were performed using the two algorithms (BDBH and OrthoMCL) implemented in GET_HOMOLOGUES v3.0, with 75% minimum coverage in BLAST pairwise alignments and E-value set at 10^−5^ [39]. We selected this consensus approach due to the robustness of the homolog clustering generated by the combination of clustering algorithms. Statistical estimation of the theoretical core- and pan-genome sizes was performed with OrthoMCL predicted gene families by fitting Willenbrock exponential model. Different compartments of the pan-genome (‘soft-core’, ‘shell’, and ‘cloud’) were also further computed using the script ‘parse pangenome matrix.pl’ included in the GET_HOMOLOGUES v3.0 [39].

In this study, we defined genome-specific as those genes detected only in one genome and absent in other genomes analyzed. We employed multiple strategies to compute the genome-specific genes in *Endozoicomonas* sp. OPT23 as illustrated in Figure S1. (*i*) Initial method involves the estimation of the genome-specific genes using the script ‘parse pangenome matrix.pl’ included in the GET_HOMOLOGUES v3.0 [39]. (*ii*) In the second method, predicted protein sequences were subjected to orthologous group inference using OrthoFinder v2.3.1 [40] as mentioned above and determined the genome-specific genes—those genes present in only one species (i.e., in *Endozoicomonas* sp. OPT23) and were unassigned to a specific orthogroup. (*iii*) In the third method, only genes shared between methods 1 and 2 were regarded as genome-specific genes (i.e., consensus data) with greater confidence. Consensus data and ‘unique’ genes solely detected by methods 1 and 2 (here after referred to as ‘outliers’) were further searched with BLASTp with the E-value set at 10^−5^ against in-house preformatted nonredundant (nr) NCBI database nr_v5 (accessed on April, 2019) restricting the search to the sequences in the database that correspond to *Endozoicomonas* (NCBI:txid305899). Sequences matching the database entries were subsequently removed from the list of genome-specific genes. The ‘outlier’ genes recovered from GET_HOMOLOGOUS were not considered for further analyses due to false positive hits. The final set of genome-specific genes was selected by combining the sequences obtained from consensus approach and manually curated ‘outlier’ genes detected by OrthoFinder.

Genome-specific genes of *Endozoicomonas* sp. OPT23 were predicted by performing BLASTp with E-value set at 10^−5^ against the locally formatted set of essential genes retrieved from the database of essential genes (DEG, http://tubic.org/deg_bak/download.php; accessed on April, 2019) [41].

### 2.6. Prediction of Symbioses Factors and Secretion Systems

The genes coding for eukaryotic-like proteins (ELPs) such as ankyrin repeats (ANKs), tetratricopeptide repeat (TPRs), and Sel1 repeat-containing proteins were searched in the annotation files using the key words ‘repeats’, ‘Ankyrin’, ‘Tetratricopeptide’, and ‘Sel1′. Furthermore, the protein sequences of the *Endozoicomonas* genomes were scanned against local InterPro’s proteins signature database using InterProScan v5.32-71.0 [42]. The output files were screened for the protein domains using InterPro entries- ANKs (IPR020683 and IPR002110), TPRs (IPR011990, IPR019734, IPR013105, IPR001440, and IPR011717), and Sel1 (IPR006597) as previously reported [7,43]. In order to avoid false prediction due to high similarity of proteins containing TPRs and Sel1 repeat, TPRpred v10.3- a profile-based tool was used to classify TPRs and Sel1 repeats within a protein using E-value cutoffs of 10^−3^ and 10^−2^ [44].

Secretion systems in the genome were screened and classified by BLAST search using the whole proteome as a query with BlastKOALA (Available online: https://www.kegg.jp/blastkoala/) tool against the taxonomic group “Bacteria” and “genus_prokaryote” database in KEGG v2.2 [45]. Bacterial genomes were further screened for type VI secretion system gene clusters by using HMM searches implemented in SecReT6 [46]. Type VI and type III secretion system effector proteins (T6Es and T3Es) were predicted using bacterial secreted effector protein database SecretEPDB [47]. Briefly, a total of 175 and 1194 T6 and T3 effector protein sequences were extracted from the SecretEPDB and the homologous protein sequences from the *Endozoicomonas* sp. OPT23 genomes were recovered using BLASTP search with an E-value cutoff of 10^−5^. FIMO v5.05 implemented in the MEME suite [48] was used to scan the proteins for the presence of motifs Tle1-4 (GxSxG) and Tle5 (HxKxxxxD).

### 2.7. Data Deposition

This project has been deposited at GenBank under the accession PPFD00000000.

## 3. Results and Discussion

### 3.1. Genome Summary and Phylogeny of the Endozoicomonas sp. OPT23

**Figure 1:**
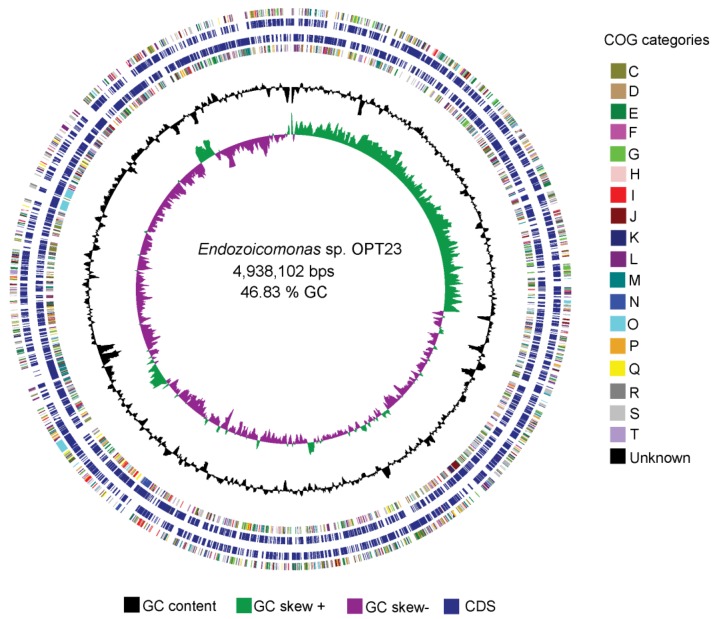
Graphical circular representation of the genome of the sponge-associated *Endozoicomonas* sp. OPT23. Circles from interior to exterior represent: (1) GC skew, (2) GC content, (4) coding sequences on forward strand, (5) coding sequences on reverse strand, and (3 and 6) clusters of orthologous groups (COG) categories.

**Figure 2:**
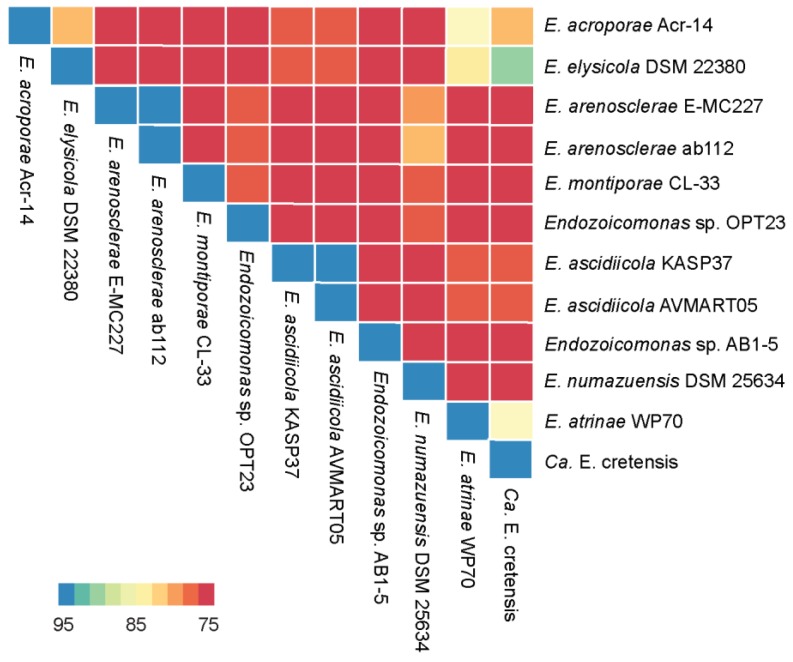
Heatmap representing the degree of similarity of the 12 Endozoicomonas genomes studied. The heatmap was derived from the average nucleotide identity (ANI) matrix based on BLAST (ANIb) approach. Color scheme varies from high similarity (blue) to low similarity (red) of the genomes analyzed (please see color range patterns and the corresponding similarity values highlighted in the bar legend of the figure).

The genome assembly and sequencing of *Endozoicomonas* sp. OPT23 isolated from the intertidal marine sponge, *O. papilla* retrieved a total of 30 scaffolds (N_50_ of >0.73 Mbp) and revealed a genome size of 4.9 Mbp with a G+C content of 46.84% (Table 1, Figure 1). The genome was nearly complete (99%) with low level of contamination (<5%), based on CheckM analysis with the Gammaproteobacteria gene marker set. The genome annotation predicted a total of 4304 genes, of which 4175 (97%) were protein-coding. Genetic relatedness estimation using ANI and DDH, and its comparison with previously sequenced genomes suggest that sponge-associated *Endozoicomonas* sp. OPT23 is a novel species (average ANIb value 75% ± 7.5% and average DDH value of 22% ± 1.03%), which are below the threshold of 95% for ANI and 70% for DDH used to delineate species (Figure 2, Table S1). Approximately 79% of the proteins could be assigned based on COGs using eggNOG database. Comparison of the proteins assigned to COGs among *Endozoicomonas* strains isolated from different habitats did not show any clear differences in the distribution of the proteins among most of the COG categories (Figure S2). Though *Endozoicomonas* sp. OPT23 represent the second smallest genome among the members of the genus *Endozoicomonas*, we detected significant over-representation (Z-test, *p* < 0.01) of COG categories responsible for ‘energy production and conversion’ (C), in *Endozoicomonas* sp. OPT23 which are likely to encode specialized strain-specific physiological functions. For instance, further analyses revealed that COG category ‘C’ consisted of coding sequences related to various enzymes such as ATP-synthases, dehydrogenases, and ABC-transporter genes indicating the possible ability towards high nutrient uptake/synthesis, which could be an adaptation mechanism of *Endozoicomonas* sp. OPT23 to thrive in nutrient poor environment. Interestingly, we observed under-representation of genes in the COG functional category ‘replication, recombination and repair’ (L) in *Endozoicomonas* sp. OPT23 when compared to other strains except in *E. elysicola* DSM 22380 (genome size of 4 Mbp). This is mainly ascribed to the presence of a smaller number of transposase genes in *Endozoicomonas* sp. OPT23. While small bacterial genome sizes may partly explain low number of transposase genes, a previous study detected enrichment of transposase genes among *Endozoicomonas* species suggesting that these bacterial strains are not undergoing genome streamlining considering the large genome sizes [19].

**Figure 3:**
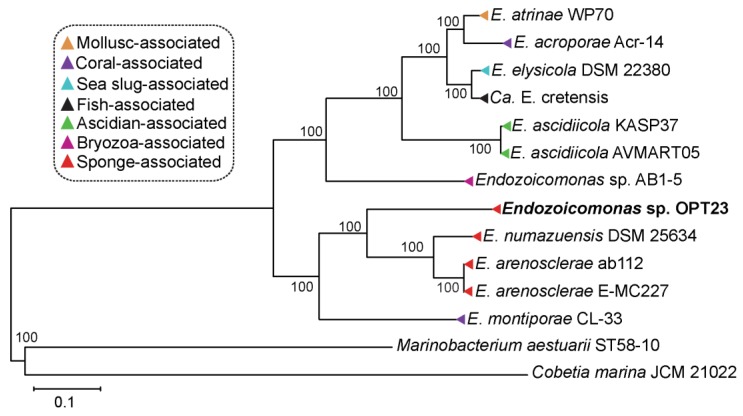
Whole genome phylogeny of the genus *Endozoicomonas*. Maximum-likelihood tree inferred using 622 single-copy orthologous genes (~209,574 sites) using LG+F+I+G4 model chosen according to BIC (Bayesian information criterion). *Endozoicomonas* strains isolated from different invertebrate hosts are labeled with different colors. Strain sequenced in this study is shown in bold. Bootstrap support values are represented at each node of the phylogenetic tree. The tree is rooted using the outgroup species *Marinobacterium aestuarii* ST58-10 and *Cobetia marina* JCM 21022.

Evolutionary relationship inference using phylogenetic approach with 622 single-copy orthologous gene sequences suggest clustering of the members of the genus *Endozoicomonas* isolated from different invertebrate hosts such as sponges (*Endozoicomonas* sp. OPT23 isolated from *O. papilla*; *E. arenosclerae* ab112, *E. arenosclerae* E-MC227 isolated from *Arenosclera brasiliensis*; and *E. numazuensis* DSM 25634 isolated from *Haliclona* sp.) and ascidian (*E. ascidiicola* AVMART05 and *E. ascidiicola* KASP37 isolated from the pharynx of *Ascidiella* sp.), might possibly suggest a co-diversification event between these hosts and symbiotic *Endozoicomonas* (Figure 3). However, other *Endozoicomonas* strains isolated from corals did not cluster together in the phylogenetic tree. The observed trend in which the bacterial species failed to cluster based on the habitat/isolation source might indicate the ability of *Endozoicomonas* bacteria to colonize and establish a symbiotic relationship with a wide variety of marine invertebrate hosts. A similar trend was previously reported among the members of the genus *Pseudovibrio* isolated from different hosts—sponges, tunicates, coral, flatworms, and free-living [6]. We hypothesize that inconsistent co-evolution of the symbiont with other invertebrate hosts might be due to the influence of other factors such as mode of transmission (horizontal vs. vertical) which warrants further investigation. Sequence information of more symbiotic *Endozoicomonas* strains might improve the phylogenetic resolution within the *Endozoicomonas* genus and to test the co-evolution hypothesis.

### 3.2. Core- and Pan-Genome of Endozoicomonas

Clustering of 61,797 CDS predicted from the 12 genomes of the genus *Endozoicomonas* derived a total of 18,704 genes defining the pan-genome. Among these, 656 genes (≈3.5%) were found in all the 12 genomes defining the core-genome (Figure S3A,B). The estimation of the core- and pan-genome sizes using Willenbrock exponential model based on orthoMCL clustering indicated a decrease in core- and an increase in pan-genome sizes. Furthermore, the pan-genome fitting curve has not reached a plateau (Figure S3C), suggesting that the pan-genome of *Endozoicomonas* is open and sequencing of additional new genomes will likely increase the gene pool size/yield novel genes.

**Figure 4:**
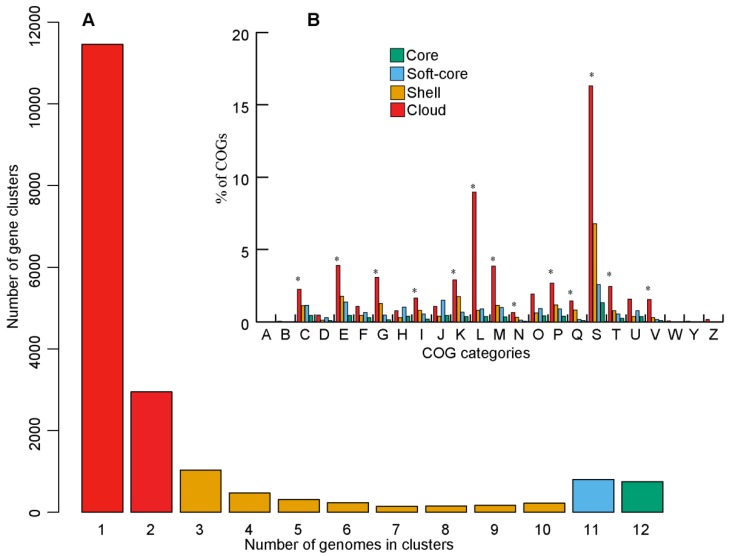
Pan-genome structureand function of the genus *Endozoicomonas*. (**A**) Bar plot representing the average frequencies of genes detected in the pan-genome. (**B**) Percentage of COGs assigned to genes detected in ‘core’, ‘soft-core’, ‘shell’, and ‘cloud’. The asterisks represent significant difference (*p* < 0.01) of COG categories detected. Error bars are not shown for visual clarity.

The evolutionary history of an organism could be inferred from the flexible genome composition analyses. Therefore, we further computed the less conserved compartments of the pan-genome structure ‘cloud’ (complement of genes present in two or fewer genomes), ‘shell’ (complement of moderately common genes present in nine genomes), and ‘soft-core’ (complement of genes present in 11 genomes) defining the flexible genome (Figure 4A). Subsets of flexible genome (‘cloud’ and ‘shell’ i.e., genes present in 2–9 genomes) were further used since they have different rates of gene acquisition and deletion [49]. Functional COG assignment revealed that majority of the COG categories were over-represented (Z-test, *p* < 0.01) in the flexible-genome relative to the core-genome, such as genes involved in ‘energy production and conversion’ (C), ‘amino acid transport and metabolism’ (E), ‘carbohydrate transport and metabolism’ (G), ‘lipid transport and metabolism’ (I), ‘transcription’ (K), ‘replication, recombination and repair’ (L), ‘cell wall/membrane/envelope biogenesis’ (M), ‘cell motility’ (N), ‘inorganic ion transport and metabolism’ (P), ‘secondary metabolism’ (Q), ‘function unknown’ (S), ‘signal transduction mechanisms’ (T), and ‘defense mechanisms’(V) (Figure 4B). This trend indicates that the less conserved flexible genomes might be responsible for the functional diversity of *Endozoicomonas* species thriving in various habitats.

### 3.3. Estimation of Endozoicomonas sp. OPT23-Specific Genes

Genome-specific genes were estimated to determine the key genomic features unique to the sponge-associated *Endozoicomonas* sp. OPT23. Approaches 1 and 2 using GET_HOMOLOGS and OrthoFinder detected 851 and 503 genome-specific genes in *Endozoicomonas* sp. OPT23 (Table S2), respectively. Observed differences in the predicted number of genome-specific genes were expected due to the differences in the similarity search algorithms used for clustering the homologous genes. The consensus method and further validation by taxonomically restricted BLASTp search against nr databases limited the ‘false positive’ identification of genes as genome-specific genes. For instance, our manual inspection and curation suggests that ≈45% of genes identified by GET_HOMOLOGUES were not genome-specific (data not shown). A final set of 506 genome-specific genes was detected in *Endozoicomonas* sp. OPT23 (Figure S1, Table S2).

Approximately 73% of the genome-specific genes in *Endozoicomonas* sp. OPT23 were predicted as hypothetical proteins. The genes encoding for functionally unknown proteins might have an important biological role [50] and hence we performed the annotation of *Endozoicomonas* sp. OPT23-specific genes using essential gene database. Only 8% of the genome-specific genes have homologs in the essential gene database and categorized to the class carbohydrate transport and metabolism, adaptation/protection, fatty acid and phospholipid metabolism, energy metabolism, and unknown function (Table S3). For instance, we identified two homologs of OsmC (osmotically inducible protein C)-like family proteins (IPR003718) known to be involved in defense against oxidative stress caused by organic hydroperoxide, a byproduct of bacterial aerobic respiration [51]. We speculate that detoxification of hydroperoxide to less toxic compounds might help *Endozoicomonas* sp. OPT23 to survive and proliferate within the host. Other major features detected in the genome of *Endozoicomonas* sp. OPT23 are discussed in the following sections.

### 3.4. Metabolic Specificity of Endozoicomonas sp. OPT23

#### 3.4.1. Lactate Utilization Pathway

**Figure 5:**
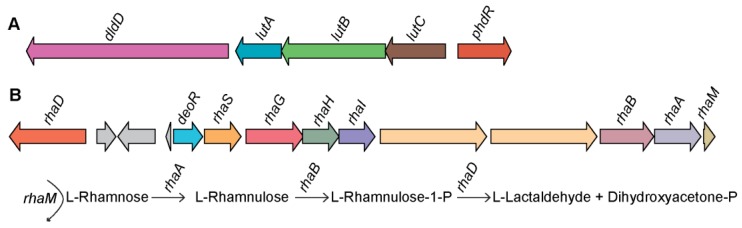
Genetic organization of the predicted lactate and L-rhamnose catabolic gene clusters in *Endozoicomonas* sp. OPT23. (**A**) Lactate catabolic gene cluster coding for D-lactate dehydrogenase (*dldD*), three iron-sulfur-containing proteins (*lutABC*), and transcriptional regulator (*phdR*). (**B**) L-rhamnose (L-Rha) utilization pathway gene cluster encoding for the enzymes L-rhamnulose-1-phosphate aldolase (*rhaD*), L-rhamnulokinase (*rhaB*), L-rhamnulose isomerase (*rhaA*), L-rhamnulose mutarotase (*rhaM*), L-Rha uptake transporter genes (*rhaSGHI*), and transcriptional regulator (*deoR*). A schematic diagram illustrating the conversion of L-Rha to the final products is also shown. Straight and curved arrows indicate the enzymatic reactions and the genes involved in each step, respectively.

Genome-specific and metabolic pathway comparison of the members of the genus *Endozoicomonas* using subsystem-based analyses implemented in RAST [52] predicted the lactate utilization genes arranged in an operon encoded for a D-lactate dehydrogenase (dldD, E.C 1.1.2.4), three iron-sulfur-containing proteins (lutABC), and a gene coding for FadR transcriptional regulator family (phdR) in the genome of *Endozoicomonas* sp. OPT23 (Figure 5A). BLASTp similarity search of the individual genes encoded in lactate regulon against the nr database detected orthologs of dldD, lutABC, and FadR (70%–90% sequence identity) in a distantly related species of *Oceanospirillales* bacterium Hp36 isolated from the Icelandic intertidal marine sponge *Halichondria panacea* and *Parendozoicomonas haliclonae* isolated from a sponge of the genus *Haliclona* (Figure S4). Lactate utilization allows microbes with the ability to use lactate (D- and/or L-lactate), a ubiquitous carbon source in nature. In microorganisms, D- or L-lactate dehydrogenase (genes *dldD* and *lldD*) play a key role in the lactate utilizing pathway through oxidation of lactate to pyruvate, which is further incorporated into the central carbon metabolism [53]. While dldD was only detected in the genome of *Endozoicomonas* sp. OPT23, seven other *Endozoicomonas* spp. genomes (including two species isolated from sponges) contained genes encoding L-lactate dehydrogenase (lldD, E.C 1.1.2.3), whereas the remaining four strains were devoid of the genes coding for either lldD or dldD. It indicates those *lldD*/*dldD* are not common genetic traits among *Endozoicomonas* spp., genomic characteristics previously reported for the genus *Streptococcus* [54]. However, the observed syntenic genetic organization of *lutABC* operon (Figure S4) is due to the conserved nature of *lutABC* operon among a wide range of bacteria [55]. The role of catabolic operons *lutABC* (*lldEFG*) in lactate utilization was studied in several microbes such as *Desulfovibrio vulgaris* [56], *Campylobacter jejuni* [57], *Bacillus subtilis* [55], and *Shewanella oneidensis* [58]. *LutABC* operons have been shown to be involved in biofilm formation [55], pathogenesis [59,60,61,62], suggesting the role of bacterial lactate metabolism in adaptation/virulence in different niches. It is likely that the orthologs of *lutA*, *lutB*, and *lutC* of *lutABC* operon mediate lactate utilization in *Endozoicomonas* sp. OPT23 and might help the bacterium to thrive within the sponge host.

#### 3.4.2. L-Rhamnose Utilization Pathway

Analyses of the genome-specific genes revealed the presence of the genes organized in a putative operon encoding the L-rhamnose (L-Rha) metabolic pathway in *Endozoicomonas* sp. OPT23. L-Rha is a deoxy-hexose sugar commonly found as a constituent of pectin/hemicellulose polysaccharides present in plants and as a bacterial cell wall component [63,64]. L-Rha is utilized as a carbon source in many microorganisms [65,66]. A study involving comparative genomics of L-Rha pathway among bacteria of diverse taxonomic groups suggests the variations in the key genes responsible for the enzymes, transporters, and regulators for L-Rha utilization [67]. For instance, our analysis using subsystem-based comparative genomics implemented in RAST [52] detected a canonical L-Rha catabolic pathway in *Endozoicomonas* sp. OPT23 comprising of genes coding for four enzymes, L-rhamnulose-1-phosphate aldolase (RhaD, EC 4.1.2.19), L-rhamnulokinase (RhaB, EC 2.7.1.5), L-rhamnulose isomerase (RhaA, EC 5.3.1.14), and L-rhamnulose mutarotase (RhaM, EC 4.1.2.19) (Figure 5B). Experimental evidence shows that these enzymes are involved in the degradation of L-Rha to dihydroxyacetone phosphate (DHAP) and L-lactaldehyde via a phosphorylated pathway in many bacteria [68,69]. Comparison of L-Rha utilization genes of *Endozoicomonas* sp. OPT23 with other well studied bacterial lineages [67] revealed a lack of a syntenic organization of the L-Rha operon, suggesting a possible new genetic architecture of L-Rha pathway genes in *Endozoicomonas* sp. OPT23. L-Rha regulon of OPT23 also encoded an ABC transporter for L-Rha uptake (*rhaSGHI*), a gene coding for a putative DeoR-type transcriptional regulator (56% sequence similarity to DeoR transcriptional regulator of *Agrobacterium tumefaciens*) and two genes coding for putative α-L-rhamnosidases (EC 3.2.1.40), which exhibited a sequence similarity (up to 51%) with proteins annotated as glycoside hydrolase (GH) 78 family from *Streptomyces cyaneus*. Transcriptional regulators belonging to DeoR family have been reported to control the L-Rha regulon in *Rhizobium leguminosarum* bv. *trifolii* [70] and in *Chloroflexus aurantiacus* [67]. Alpha-L-rhamnosidases catalyze the hydrolysis of α-L-rhamnosyl-linkages in L-rhamnosides present in polysaccharides. We speculate that *Endozoicomonas* sp. OPT23 isolated from a sponge might have the ability to catabolize L-rhamnose and use it as a carbon source.

#### 3.4.3. Pheynylacetic Acid Degradation Pathway

**Figure 6:**
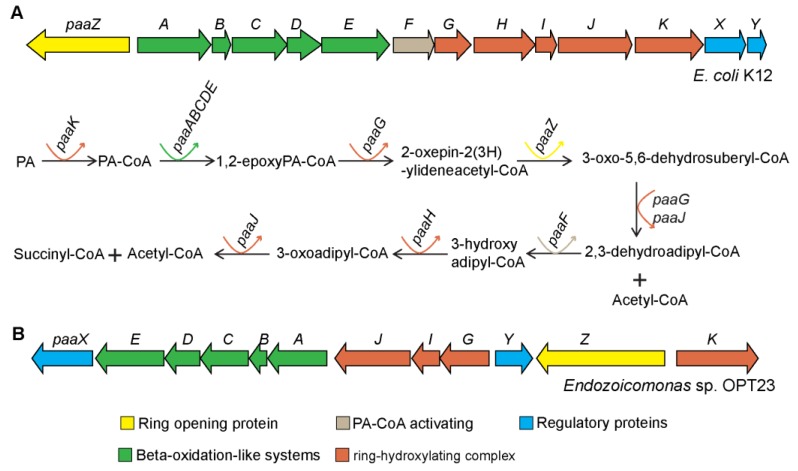
Genetic organization of phenylacetate degradation pathway gene clusters. (**A**) *Escherichia coli* K12 encoded phenylacetate catabolic pathway gene cluster and suggested reactions and intermediates of the pathway [71,72]. (**B**) Predicted putative phenylacetate degradation pathway gene clusters in the genome of *Endozoicomonas* sp. OPT23. Genes are color coded according to the reaction steps involved in the phenylacetate catabolism. Straight and curved arrows indicate the enzymatic reactions and the genes involved in each step, respectively.

A gene cluster (≈11Kbp) with a total of 12 genes related to the phenylacetic acid (PA) catabolic pathway was exclusively detected in the genome of *Endozoicomonas* sp. OPT23 (Figure 6). Metabolism of phenylacetic acid (phenylacetate) is a central metabolic route of several aromatic compounds derived from substrates such as phenylalanine, liginin-related phenylpropane units, 2-phenylethylamine, phenylacetaldehyde, or environmental pollutants such as styrene and ethylbenzene, into a common intermediate, phenylacetyl-CoA, which is subsequently fed to the Krebs cycle [71,72]. Genomes of environmentally important aromatic degrading microbes such as *Escherichia coli* [73], *Pseudomonas putida* [71], and several members of the genus *Roseobacter* [74] were reported to harbor PA catabolic gene clusters organized in several contiguous operons consisting up to 17 genes. Although not enough evidence is available to validate the functional roles of the gene products encoded in the PA gene clusters of many microbes, some experimental studies validated the possible functions of the PA degradation pathway genes [72,75,76]. For instance, paaK (phenylacetyl-coenzyme A (PA-CoA) ligase) is responsible for the activation of phenylacetate to phenylacetyl-CoA, the first common intermediate of PA pathway. Five-component oxygenase- paaA, paaB, paaC, paaD, and paaE catalyze the 1,2-epoxidation of PA-CoA. The PA-CoA ring hydroxylation system, comprised of paaG, paaZ, and paaJ, followed by a β-oxidation-like pathway complex by paaH, paaF, and paaJ. The PA gene cluster also encodes for a transcriptional repressor paaX, and a putative regulator protein encoding gene paaY.

Our comparative genomics analysis revealed that the genes of the PA catabolon of the sponge-associated *Endozoicomonas* sp. OPT23 are organized as a single operon, *paaXEDCBAJIGYZK* and are syntenic with the PA gene cluster of *Paraglaciecola agarilytica* NO2 belonging to the order *Alteromonadales* (Figure S5). We detected a discrepancy in the annotation of *paaJ*, which was annotated as a homologous gene *pcaF* coding for β-ketoadipyl-CoA thiolase. Subsequent BLASTp analysis suggest a sequence similarity of *pcaF* with *paaJ* which also codes for β-ketoadipyl-CoA thiolase of *E. coli* (E-value 0.0, percentage identity 71%), a trend/classification previously reported in the pathogenic bacterium *Burkholderia cenocepacia* [75]. It is intriguing that the PA catabolon pathway gene cluster was not detected in any other members of the genus *Endozoicomonas* analyzed in this study indicating that *Endozoicomonas* sp. OPT23 might have the ability to utilize phenylacetate as a carbon source and might contribute towards the virulence for the survival within the sponge hosts. Probable links between bacterial virulence and phenylacetate catabolism have been proposed in *Mycobacterium abscessus* and *B. cenocepacia* [75,77]. In addition to the utilization of phenylacetate generated through the degradation of aromatic amino acids in proteins, we argue that *Endozoicomonas* sp. OPT23 might metabolize phenylacetate released from the breakdown of liginin present in algae suggesting that *Endozoicomonas* sp. OPT23 could be an opportunistic symbiont of the sponge.

The several physiological traits mentioned above encoded in the genome of *Endozoicomonas* sp. OPT23 could be validated in future using laboratory experiments.

### 3.5. Symbioses Factors and Secretion Systems

#### 3.5.1. Symbioses-Related Genes in *Endozoicomonas* sp. OPT23

The genome of the sponge-associated *Endozoicomonas* sp. OPT23 was encoded with the genes coding for ELPs like ANKs, TPRs, and Sel1-like domain containing proteins (Table S4). These symbioses-related genes, present in pathogenic and symbiotic microbes, are reported to mediate the intracellular survival and pathogenicity by interfering with eukaryotic protein–protein interactions [78,79]. Abundance of ELPs seems to be a major genomic feature of sponge symbionts [3,4,80]. Our recent study reported that the genome of *Shewanella* sp. OPT22 and *S. spongiae* KCTC 22492 isolated from sponges are enriched with genes coding for ANKs when compared to other *Shewanella* strains inhabiting other niches [7]. The role of ANKs from sponge symbionts in modulating the amoebal phagocytosis was experimentally validated [81]. Furthermore, comparative genomics analyses revealed the presence of all three ELPs in varying proportions (in a range of 1–111ANKs, 23–30 TPRs, and 1–10 Sel1) in the genomes of the members of the genus *Endozoicomonas* examined here (Figure S6). It is not surprising to observe these genome features due to the fact that the *Endozoicomonas* species examined in this study either have symbiotic lifestyle such as in sponges, corals, ascidians, mollusks, sea slugs, bryozoa, or pathogenic lifestyle such as in fishes. Intriguingly, we detected an overabundance of ANKs (*n* = 111) in the genome of *E. acroporae* Acr14 isolated from a coral (*Acropora*). Closer inspection of the genome of *E. acroporae* Acr14 revealed that detected genes are true ANKs and ruled out the possibility of false prediction of repeat regions at the contig boundaries. Overall, it is clear that detected ELPs might play a crucial role in establishing either symbiotic or pathogenic association between *Endozoicomonas* and various invertebrate hosts.

#### 3.5.2. Role of Secretion Systems in Host-Bacterial Relationship

Bacteria use a variety of dedicated secretion systems (SSs) in order to transport protein cargos across their membrane and cell wall for interacting with the environment to establish a favorable niche. Here, we focused only on some of the secretions systems like T3SSs and T6SSs (i.e., components of the type III and type IV secretion system, respectively) which are the key players in establishing a host-associated lifestyle.

**Figure 7:**
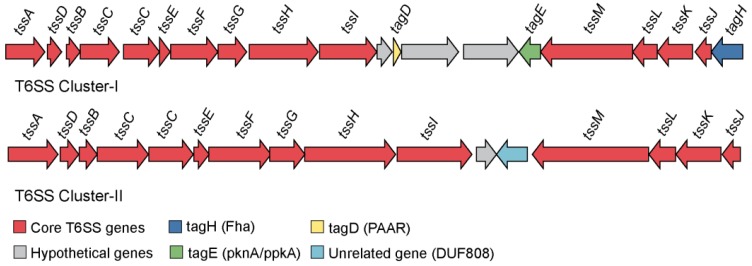
Genetic organization of the annotated type VI secretion system gene clusters in *Endozoicomonas* sp. OPT23. Two clusters representing genes coding for predicted type VI secretion system (T6SS) apparatus. Genes are represented by colored arrows and gene names are given above the arrows according to the tss nomenclature. Red colored arrows identify the core T6SS apparatus, dark blue and yellow arrows represent the genes coding for Fha and PAAR domains, respectively, genes coding for hypothetical proteins are shown in grey, green arrows represent tagE and light blue denote an unrelated gene within the T6SS gene cluster.

Homologs of the T6SS were clustered in two distinct genomic regions (contigs) of *Endozoicomonas* sp. OPT23 sequenced in this study. These regions were designated as T6SS-I (26 Kbp) and T6SS-II (21 Kbp) (Figure 7), composed of core components—Tss (type VI secretion system) and PAAR, Tag (type VI secretion system associated) proteins with structural, regulatory or effector functions. Furthermore, the T6SS-I and T6SS-II system of *Endozoicomonas* sp. OPT23 has 20 and 16 genes in common with the sponge-associated *Oceanospirillales* bacterium Hp36, representing 14 and 13 core genes of the T6SS-I and T6SS-II cluster, respectively. Further, they share synteny in core T6SS genes (Figure S7). T6SS play an important role in protein secretion across their envelope and injecting the toxic effector proteins by direct contact into other prokaryotes or delivered into the eukaryotic hosts to gain access to the resources and dominate in a specific niche [82,83,84,85]. T6SS have been reported to be involved in host–pathogen interactions, bacterial predation and inter/intraspecific competition, bacterial communication, biofilm formation, and symbioses [86,87]. T6SS detected in the *Endozoicomonas* sp. OPT23 might be functional due to the presence of all structural components of the T6SS apparatus.

We further screened the genome of the *Endozoicomonas* sp. OPT23 for the presence of potential type VI effector proteins (T6Es) (Table S5). The most common proteins with effector functions, such as Hcp (haemolysin coregulated proteins), VgrG (valine-glycine repeat protein G), and PAAR (proline-alanine-alanine-arginine) repeat proteins were detected within the T6SS gene clusters. Interestingly, we did not find any ‘orphan’ VgrG or Hcp genes in the sequenced genome of *Endozoicomonas* sp. OPT23. Besides the structural role, VgrG proteins participate in the disruption of the actin cytoskeleton, T6SS-mediated intracellular competition, and host–microbe interactions [88]. It is known that most of the VgrG proteins involved in effector functions present specific extended C-terminal functional domains. However, with our approach we could not identify the presence of such domains in the proteins of the *Endozoicomonas* sp. OPT23. Lack of homologous VgrG genes in the genome and absence of effector functional domains in VgrG genes indicates that VgrG genes of *Endozoicomonas* sp. OPT23 might play a structural role. The genome of *Endozoicomonas* sp. OPT23 encoded one potential effector protein belonging to type VI lipase effectors (Tle). The Tle proteins are T6SS phospholipase effectors involved in targeting the bacterial cell membrane by hydrolyzing the lipid component [89]. Scanning of Tle protein predicted in the *Endozoicomonas* sp. OPT23 genome using FIMO revealed the presence of GxSxG motif, further categorizing the Tle protein to Tle1–4 families. We speculate that the Tle effector might help the sponge-associated *Endozoicomonas* sp. OPT23 to compete with other bacterial communities for survival within the sponge host by targeting the cell wall membrane of prokaryotic competitors. In addition, we detected the homologues of signal peptide-containing RbsB (ribose-binding protein)-like effector protein encoding genes in *Endozoicomonas* sp. OPT23. RbsB is a periplasmic ribose-binding protein encoded in the loci of ribose transport [90] and involved in the binding of substrates such as ribose or autoinducer 2 (AI-2) signaling molecules [91]. Though the relationship between the RbsB-like proteins and T6SS, and its possible role in symbioses are not clear, RbsB-like proteins have been reported as a T6SS substrate in symbiotic *Rhizobium leguminosarum* [92]. We speculate that the RbsB-like proteins might play a role in enhancing the fitness of *Endozoicomonas* sp. OPT23 within the eukaryotic sponge hosts. Further experimental evidence is required to confirm the role of the above mentioned T6Es in symbiosis with sponges.

In addition to T6SS, we identified a T3SS in the genome of *Endozoicomonas* sp. OPT23 (Figure S8). It was located in different genomic regions as gene clusters encoding highly conserved genes responsible for T3SS apparatus. T3SS is a major genetic determinant of Gram-negative bacteria that facilitate the interaction between bacteria and eukaryotic hosts [93]. Besides secretion system apparatus genes encoding homologues of several effector molecules were also detected in the genome of *Endozoicomonas* sp. OPT23 (Table S5). For instance, type III effector proteins YopH (Yersinia outer protein), IpgD (inositol phosphate phosphatase), HopI1, HopJ1 (Hrp outer protein), and PipB2 (Pathogenicity island-encoded protein) were reported to act as virulence factors to enhance the proliferation of bacterial cells after attachment with eukaryotic hosts [94], help the bacterial invasion and dissemination [95], and recruitment of kinesin-1 on the Salmonella-containing vacuole (SCV), for maintaining a pathogenic lifestyle [96]. Presence of the genes encoding the secretion systems and the effector proteins in the genome of *Endozoicomonas* sp. OPT23 suggest the ability of the sponge-associated bacterium to interact with the eukaryotic sponge and live in a symbiotic relationship.

## 4. Conclusions

The bacterial members of the recently described genus *Endozoicomonas* are ubiquitous and frequently associate with diverse marine hosts. Several studies reported the genome sequences of *Endozoicomonas* strains isolated from various invertebrate hosts, but limited information is available on the genomic features of the sponge-associated *Endozoicomonas*. In this study, we sequenced the genome of *Endozoicomonas* sp. OPT23 isolated from the intertidal marine sponge *O*. *papilla* to gain further insight into the genomic architecture of the sponge-associated *Endozoicomonas* sp. OPT23 and the molecular mechanisms involved in establishing a successful association with the marine sponge. Though our genus-wide comparative genomics analyses revealed a general trend of uniformity at a functional level, genome-specific analyses suggest the presence of gene clusters encoding for the lactate, L-rhamnose metabolism, and phenylacetic acid (PA) degradation pathway indicating the probable ability of *Endozoicomonas* sp. OPT23 to utilize the alternative carbon sources. In addition to the observed metabolic specificity, the genome was encoded for eukaryotic-like proteins, which might favor the bacterium to evade the hosts’ immune response and survive within the host cell. Furthermore, the major secretion system machineries were also detected in *Endozoicomonas* sp. OPT23 that might facilitate in delivering the virulence molecules Tle/RbsB-like effector protein across the bacterial membrane to the sponge host. Conclusively, the genome sequenced in this study represents an important addition to the less represented recently described genus *Endozoicomonas* and shed further insight into the sponge–bacteria interactions.

## Figures and Tables

**Table 1:** List of organisms used for comparative study.

Organism	Habitat	Bioproject	Assembly Version	# Contigs	Genome Size (bp)	%G+C Content	# Genes	# CDS	%COGs
*Endozoicomonas* sp. OPT23 *	Sponge	PRJNA430358	ASM965363v1	30	4,938,102	46.84	4304	4175	79.49
*E. arenosclerae* ab112	Sponge	PRJNA279233	ASM156201V1	328	6,453,554	47.65	5752	5571	72.15
*E. arenosclerae* E-MC227	Sponge	PRJNA279233	ASM156200v1	2501	6,216,773	47.15	6025	5874	61.52
*E. numazuensis* DSM 25634	Sponge	PRJNA224116	ASM72263v1	31	6,342,227	47.02	5647	5468	70.90
*E. ascidiicola* AVMART05	Ascidian	PRJNA291958	AVMART05_1.0	36	6,130,497	46.70	5423	5282	68.61
*E. ascidiicola* KASP37	Ascidian	PRJNA291960	KASP37_1.0	34	6,512,467	46.65	5768	5629	66.69
*E. montiporae* CL-33	Coral	PRJNA66389	ASM158343v1	1	5,430,256	48.46	5125	4935	74.59
*E. acroporae* Acr14	Coral	PRJNA422318	ASM286404v1	309	6,048,850	49.16	5274	5015	64.85
*Endozoicomonas* sp. AB1-5	Bryozoa	PRJNA322176	-NA-	272	4,049,356	45.28	3694	3525	78.27
*E. elysicola* DSM 22380	Sea slug	PRJNA252578	ASM71077v1	2	5,606,375	46.75	4785	4652	76.13
*Ca.* E. cretensis	Fish	PRJEB7440	-NA-	638	5,876,352	46.80	5641	5505	68.30
*E. atrinae* WP70	Bivalve	PRJNA224116	-NA-	980	6,687,418	47.94	6288	6166	65.65

* Genome sequenced in this study. ^#^ Number of.

## Supplementary Materials

The following are available online at https://www.mdpi.com/2076-2607/7/12/635/s1, Figure S1: Schematic layout of workflow adopted for estimating the genome-specific genes in *Endozoicomonas* sp. OPT23, Figure S2: Heatmap representation absolute frequency of the clusters of orthologous groups (COG) of proteins assigned to the members of the genus *Endozoicomonas*, Figure S3: Pan- and core-genome structure of the genus *Endozoicomonas*. Venn diagram representing the consensus (A) pan- and (B) core-genome clusters estimated using COG and OMCL clustering algorithms. Statistical estimation of (C) the pan- and (D) core-genome sizes of the genus *Endozoicomonas*, Figure S4: Comparison of lactate catabolic gene clusters, Figure S5: Comparison of phenylacetate catabolon detected in *Endozoicomonas* sp. OPT23 and *Paraglaciecola agarilytica* NO2, Figure S6:A bar graph showing the distribution of predicted genes-containing eukaryotic-like proteins (ELPs) in the genus *Endozoicomonas*, Figure S7: Genetic organization of two type VI secretion system gene clusters detected in *Endozoicomonas* sp. OPT23 and its syntenic arrangement with *Oceanospirillales* bacterium Hp36, Figure S8: Genetic organization of type III secretion system detected in *Endozoicomonas* sp. OPT23 and its syntenic arrangement with *Oceanospirillales* bacterium Hp36, Table S1: (A) Average nucleotide identity (ANI) values calculated for 12 *Endozoicomonas* species. (B) *In silico* DNA-DNA hybridization (DDH) estimate using *Endozoicomonas* sp. OPT23 as a query, Table S2: List of genome-specific genes (locus tag) detected in the genome of *Endozoicomonas* sp. OPT23 by GET_HOMOLOGS, OrthoFinder, and manual methods, Table S3: List of essential genes predicted within the genome-specific genes of *Endozoicomonas* sp. OPT23, Table S4: List of eukaryotic-like proteins predicted in the genome of *Endozoicomonas* sp. OPT23, Table S5: List of predicted type VI secretion system effectors (T6Es) and type III secretion system effectors (T3Es) in the genome of *Endozoicomonas* sp. OPT23.
