# Supplementary material for: Comparative Genomics Reveals Metabolic Specificity of Endozoicomonas Isolated from a Marine Sponge and the Genomic Repertoire for Host-Bacteria Symbioses
Source: Microorganisms. 2019 Nov 30;7(12):635. doi: 10.3390/microorganisms7120635 (PMC6955870; doi:10.3390/microorganisms7120635)

**Supplementary fig. S1** Schematic layout of workflow adopted for estimating the genome-specific genes in *Endozoicomonas* sp. OPT23


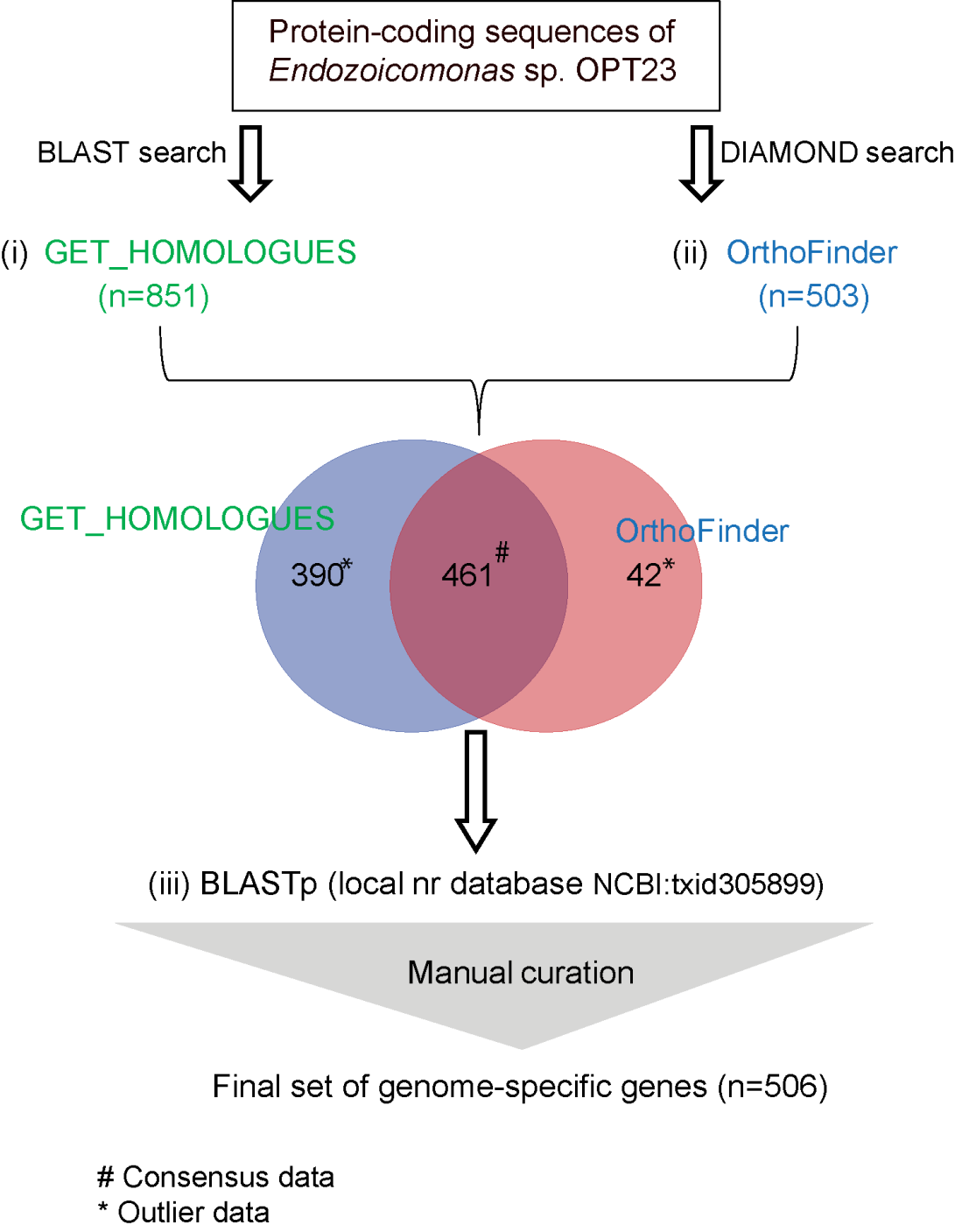

Supplement: Supplementary file 1 [file microorganisms-07-00635-s001.zip › supplementaryMaterials/FigS1.docx]
