# Supplementary material for: Comparative Genomics Reveals Metabolic Specificity of Endozoicomonas Isolated from a Marine Sponge and the Genomic Repertoire for Host-Bacteria Symbioses
Source: Microorganisms. 2019 Nov 30;7(12):635. doi: 10.3390/microorganisms7120635 (PMC6955870; doi:10.3390/microorganisms7120635)
Supplement: Supplementary file 1 [file microorganisms-07-00635-s001.zip › supplementaryMaterials/FigS2.docx]

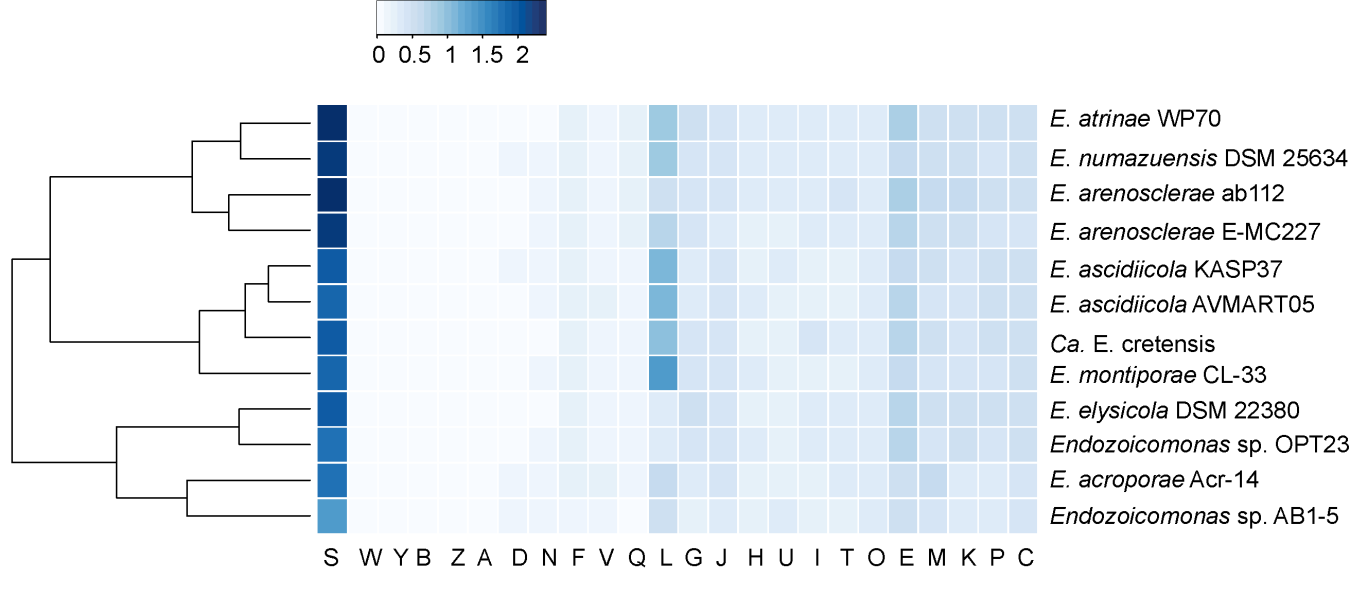


**Supplementary fig. S2** Heatmap representation absolute frequency of the clusters of orthologous groups (COG) of proteins assigned to the members of the genus *Endozoicomonas*. Light blue to dark blue represent distribution of low to high frequency of COGs in each genome.
