# Supplementary material for: Comparative Genomics Reveals Metabolic Specificity of Endozoicomonas Isolated from a Marine Sponge and the Genomic Repertoire for Host-Bacteria Symbioses
Source: Microorganisms. 2019 Nov 30;7(12):635. doi: 10.3390/microorganisms7120635 (PMC6955870; doi:10.3390/microorganisms7120635)
Supplement: Supplementary file 1 [file microorganisms-07-00635-s001.zip › supplementaryMaterials/FigS3.docx]

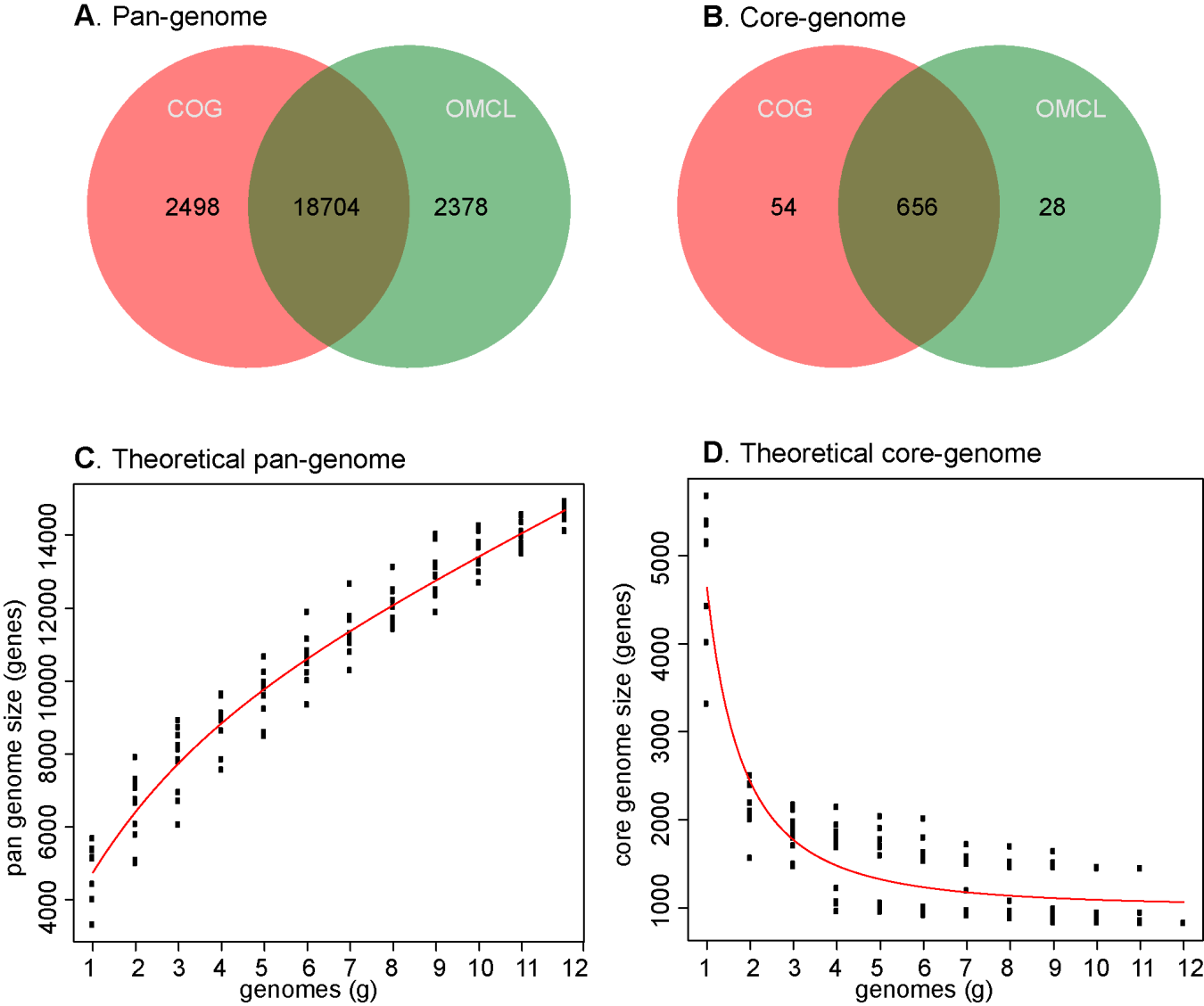


**Supplementary fig. S3** Pan- and core-genome structure of the genus *Endozoicomonas*. Venn diagram representing the consensus (A) pan- and (B) core-genome clusters estimated using COG and OMCL clustering algorithms. Statistical estimation of (C) the pan- and (D) core-genome sizes of the genus *Endozoicomonas*.
