# Supplementary material for: Comparative Genomics Reveals Metabolic Specificity of Endozoicomonas Isolated from a Marine Sponge and the Genomic Repertoire for Host-Bacteria Symbioses
Source: Microorganisms. 2019 Nov 30;7(12):635. doi: 10.3390/microorganisms7120635 (PMC6955870; doi:10.3390/microorganisms7120635)
Supplement: Supplementary file 1 [file microorganisms-07-00635-s001.zip › supplementaryMaterials/FigS4.docx]

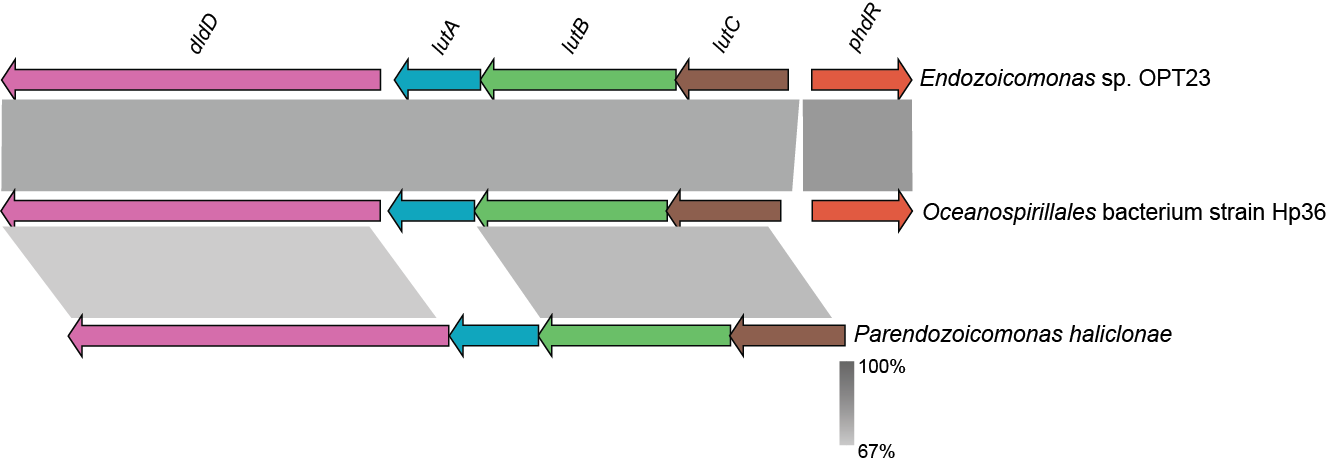


**Supplementary fig. S4** Comparison of lactate catabolic gene clusters. Genes within the cluster D-lactate dehydrogenase (*dldD*), three iron-sulfur-containing proteins (*lutABC*), and transcriptional regulator (*phdR*) are shown in unique color. For clarity, the orientations of lactate catabolic gene clusters encoded by other sponge-associated strains *Oceanospirillales* bacterium Hp36 and *Parendozoicomonas* *haliclonae* were reversed for clarity. Regions of similarity are denoted by light grey (~67%) and dark grey (~100%).
