# Supplementary material for: Comparative Genomics Reveals Metabolic Specificity of Endozoicomonas Isolated from a Marine Sponge and the Genomic Repertoire for Host-Bacteria Symbioses
Source: Microorganisms. 2019 Nov 30;7(12):635. doi: 10.3390/microorganisms7120635 (PMC6955870; doi:10.3390/microorganisms7120635)
Supplement: Supplementary file 1 [file microorganisms-07-00635-s001.zip › supplementaryMaterials/FigS5.docx]

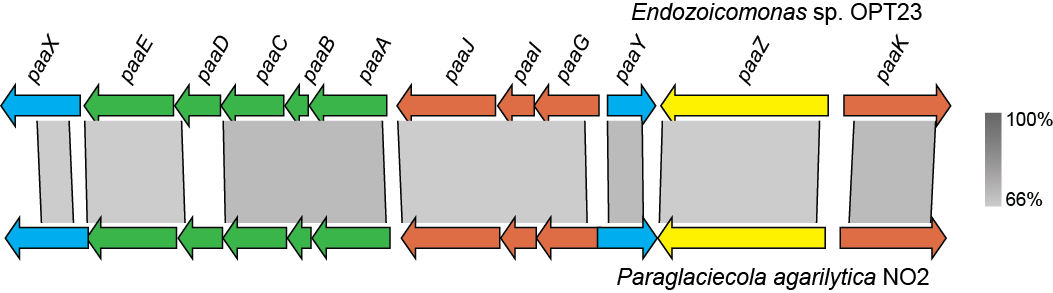


**Supplementary fig. S5** Comparison of phenylacetate catabolon detected in *Endozoicomonas* sp. OPT23 and *Paraglaciecola agarilytica* NO2. Genes detected within the gene cluster are color coded. . Regions of similarity are denoted by light grey (~66%) and dark grey (~100%).
