# Supplementary material for: Comparative Genomics Reveals Metabolic Specificity of Endozoicomonas Isolated from a Marine Sponge and the Genomic Repertoire for Host-Bacteria Symbioses
Source: Microorganisms. 2019 Nov 30;7(12):635. doi: 10.3390/microorganisms7120635 (PMC6955870; doi:10.3390/microorganisms7120635)
Supplement: Supplementary file 1 [file microorganisms-07-00635-s001.zip › supplementaryMaterials/FigS6.docx]

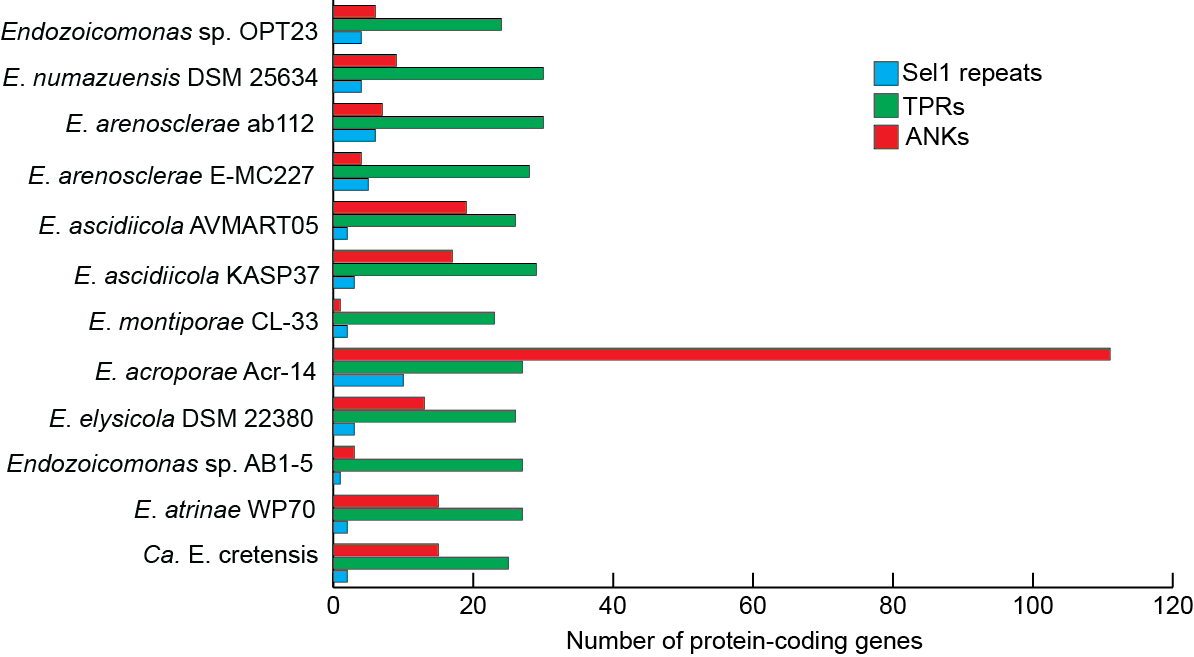


**Supplementary fig. S6**. A bar graph showing the distribution of predicted genes-containing eukaryotic-like proteins (ELPs) in the genus *Endozoicomonas*. Predicted ELPs such as ankyrin repeats- (ANKs), tetratrico repeat- (TPRs), and Sel1 repeat- containing proteins are shown in different colors.
