# Supplementary material for: Comparative Genomics Reveals Metabolic Specificity of Endozoicomonas Isolated from a Marine Sponge and the Genomic Repertoire for Host-Bacteria Symbioses
Source: Microorganisms. 2019 Nov 30;7(12):635. doi: 10.3390/microorganisms7120635 (PMC6955870; doi:10.3390/microorganisms7120635)
Supplement: Supplementary file 1 [file microorganisms-07-00635-s001.zip › supplementaryMaterials/FigS7.docx]

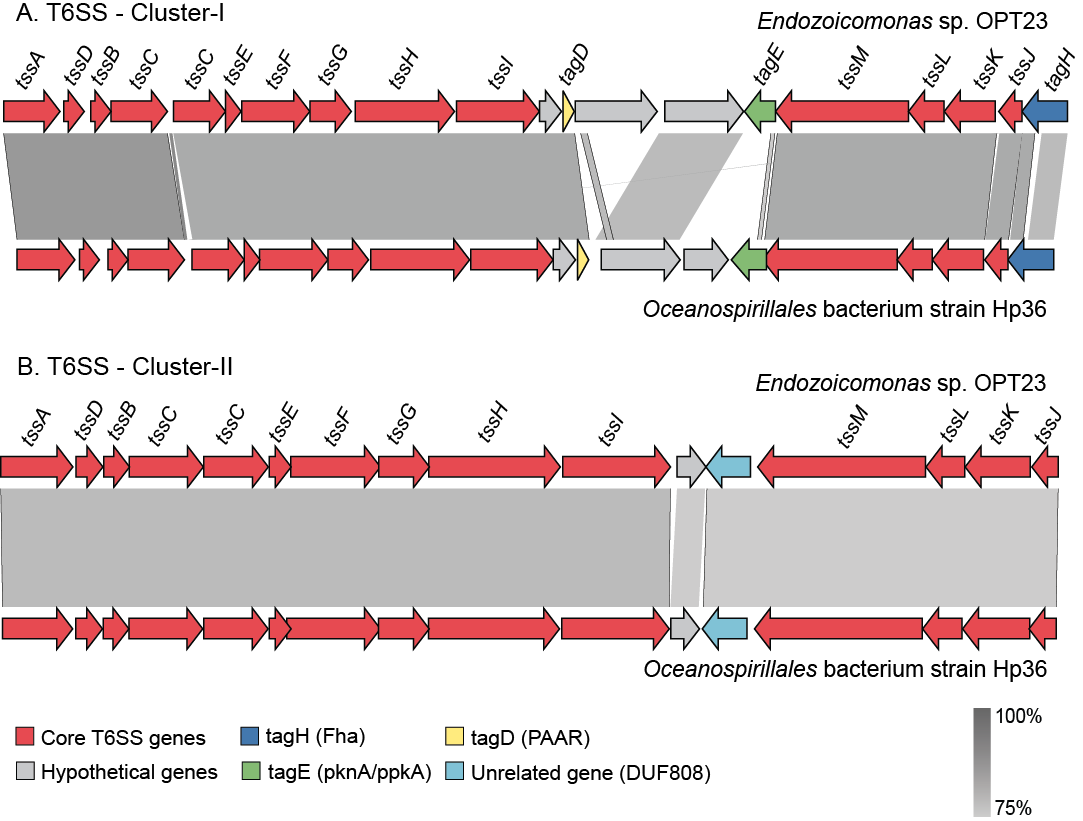


**Supplementary fig. S7**. Genetic organization of two type VI secretion system gene clusters detected in *Endozoicomonas* sp. OPT23 and its syntenic arrangement with *Oceanospirillales* bacterium Hp36. Genes are represented by colored arrows and gene names are given above the arrows according to the tss nomenclature. Red colored arrow show core T6SS apparatus, dark blue and yellow arrows represent the genes coding for Fha and PAAR domains respectively, genes coding for hypothetical proteins are shown in grey, green arrows represent tagE and light blue represent detected unrelated genes within the T6SS gene cluster. Percentage similarities of T6SS detected in the two genomes are shown in a gradient of grey color.
