# Supplementary material for: Comparative Genomics Reveals Metabolic Specificity of Endozoicomonas Isolated from a Marine Sponge and the Genomic Repertoire for Host-Bacteria Symbioses
Source: Microorganisms. 2019 Nov 30;7(12):635. doi: 10.3390/microorganisms7120635 (PMC6955870; doi:10.3390/microorganisms7120635)
Supplement: Supplementary file 1 [file microorganisms-07-00635-s001.zip › supplementaryMaterials/FigS8.docx]

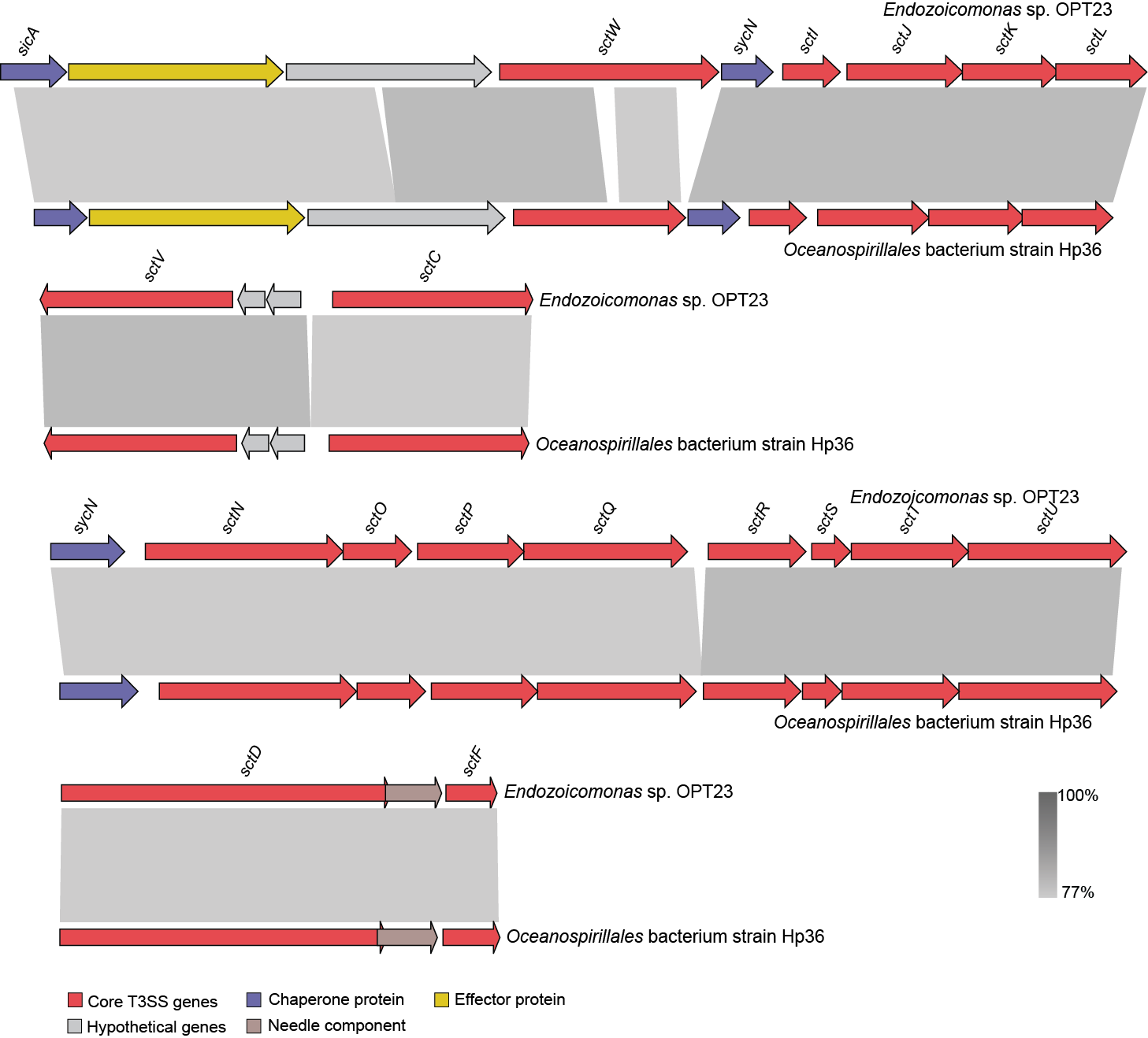


**Supplementary fig. S8**. Genetic organization of type III secretion system detected in *Endozoicomonas* sp. OPT23 and its syntenic arrangement with *Oceanospirillales* bacterium Hp36. Genes are represented by colored arrows and gene names are given above the arrows according to the sct nomenclature. Red colored arrow show core T3SS apparatus, blue arrows represent chaperone proteins, needle component genes are shown in brown color, genes coding for hypothetical proteins are shown in grey, and type III effector (T3Es) are represented in yellow color. Percentage similarities of T3SS present in two genomes are shown in a gradient of grey color.
