# Supplementary material for: Comparative Genomics Reveals Metabolic Specificity of Endozoicomonas Isolated from a Marine Sponge and the Genomic Repertoire for Host-Bacteria Symbioses
Source: Microorganisms. 2019 Nov 30;7(12):635. doi: 10.3390/microorganisms7120635 (PMC6955870; doi:10.3390/microorganisms7120635)
Supplement: Supplementary file 1 [file microorganisms-07-00635-s001.zip › supplementaryMaterials/TableS2.docx]

**Supplementary Table S2.** List of genome-specific genes (locus tag) detected in the genome of *Endozoicomonas* sp. OPT23 by GET_HOMOLOGS, OrthoFinder, and manual methods.

| **GET_HOMOLOGUES**  **(n=851)** | **OrthoFinder**  **(n=503)** | **Consenus**  **(n=461)** | **Curated**  **(n=506)** |
| --- | --- | --- | --- |
| END23_00009 | END23_00009 | END23_00009 | END23_00009 |
| END23_00012 | END23_00018 | END23_00018 | END23_00018 |
| END23_00013 | END23_00019 | END23_00019 | END23_00019 |
| END23_00014 | END23_00047 | END23_00047 | END23_00047 |
| END23_00016 | END23_00062 | END23_00062 | END23_00062 |
| END23_00018 | END23_00063 | END23_00063 | END23_00063 |
| END23_00019 | END23_00064 | END23_00064 | END23_00064 |
| END23_00027 | END23_00065 | END23_00065 | END23_00065 |
| END23_00039 | END23_00066 | END23_00066 | END23_00066 |
| END23_00043 | END23_00072 | END23_00072 | END23_00072 |
| END23_00044 | END23_00078 | END23_00078 | END23_00078 |
| END23_00047 | END23_00079 | END23_00079 | END23_00079 |
| END23_00062 | END23_00085 | END23_00085 | END23_00085 |
| END23_00063 | END23_00089 | END23_00089 | END23_00089 |
| END23_00064 | END23_00097 | END23_00101 | END23_00097 |
| END23_00065 | END23_00101 | END23_00105 | END23_00101 |
| END23_00066 | END23_00105 | END23_00109 | END23_00105 |
| END23_00067 | END23_00109 | END23_00112 | END23_00109 |
| END23_00072 | END23_00112 | END23_00122 | END23_00112 |
| END23_00078 | END23_00122 | END23_00129 | END23_00122 |
| END23_00079 | END23_00129 | END23_00132 | END23_00128 |
| END23_00085 | END23_00132 | END23_00134 | END23_00129 |
| END23_00089 | END23_00134 | END23_00140 | END23_00131 |
| END23_00096 | END23_00140 | END23_00143 | END23_00132 |
| END23_00101 | END23_00141 | END23_00144 | END23_00134 |
| END23_00104 | END23_00143 | END23_00166 | END23_00136 |
| END23_00105 | END23_00144 | END23_00167 | END23_00140 |
| END23_00109 | END23_00166 | END23_00171 | END23_00141 |
| END23_00112 | END23_00167 | END23_00175 | END23_00142 |
| END23_00119 | END23_00171 | END23_00199 | END23_00143 |
| END23_00121 | END23_00172 | END23_00212 | END23_00144 |
| END23_00122 | END23_00175 | END23_00213 | END23_00166 |
| END23_00128 | END23_00199 | END23_00229 | END23_00167 |
| END23_00129 | END23_00212 | END23_00245 | END23_00171 |
| END23_00131 | END23_00213 | END23_00249 | END23_00172 |
| END23_00132 | END23_00229 | END23_00250 | END23_00175 |
| END23_00134 | END23_00245 | END23_00257 | END23_00199 |
| END23_00136 | END23_00249 | END23_00278 | END23_00212 |
| END23_00138 | END23_00250 | END23_00283 | END23_00213 |
| END23_00140 | END23_00257 | END23_00288 | END23_00229 |
| END23_00142 | END23_00278 | END23_00289 | END23_00245 |
| END23_00143 | END23_00283 | END23_00292 | END23_00249 |
| END23_00144 | END23_00288 | END23_00300 | END23_00250 |
| END23_00162 | END23_00289 | END23_00316 | END23_00257 |
| END23_00165 | END23_00292 | END23_00318 | END23_00278 |
| END23_00166 | END23_00299 | END23_00320 | END23_00283 |
| END23_00167 | END23_00300 | END23_00336 | END23_00288 |
| END23_00171 | END23_00316 | END23_00337 | END23_00289 |
| END23_00175 | END23_00318 | END23_00360 | END23_00292 |
| END23_00199 | END23_00320 | END23_00371 | END23_00299 |
| END23_00200 | END23_00336 | END23_00374 | END23_00300 |
| END23_00212 | END23_00337 | END23_00385 | END23_00316 |
| END23_00213 | END23_00360 | END23_00396 | END23_00318 |
| END23_00229 | END23_00371 | END23_00404 | END23_00320 |
| END23_00236 | END23_00374 | END23_00405 | END23_00336 |
| END23_00243 | END23_00385 | END23_00411 | END23_00337 |
| END23_00245 | END23_00396 | END23_00438 | END23_00360 |
| END23_00247 | END23_00404 | END23_00452 | END23_00371 |
| END23_00249 | END23_00405 | END23_00459 | END23_00374 |
| END23_00250 | END23_00411 | END23_00462 | END23_00385 |
| END23_00252 | END23_00438 | END23_00464 | END23_00396 |
| END23_00254 | END23_00450 | END23_00494 | END23_00404 |
| END23_00257 | END23_00451 | END23_00497 | END23_00405 |
| END23_00278 | END23_00452 | END23_00500 | END23_00411 |
| END23_00283 | END23_00453 | END23_00512 | END23_00438 |
| END23_00284 | END23_00459 | END23_00517 | END23_00450 |
| END23_00288 | END23_00462 | END23_00519 | END23_00451 |
| END23_00289 | END23_00464 | END23_00528 | END23_00452 |
| END23_00290 | END23_00494 | END23_00535 | END23_00453 |
| END23_00292 | END23_00497 | END23_00551 | END23_00459 |
| END23_00294 | END23_00500 | END23_00569 | END23_00462 |
| END23_00295 | END23_00512 | END23_00573 | END23_00464 |
| END23_00296 | END23_00517 | END23_00574 | END23_00494 |
| END23_00297 | END23_00519 | END23_00575 | END23_00497 |
| END23_00300 | END23_00528 | END23_00578 | END23_00500 |
| END23_00316 | END23_00529 | END23_00580 | END23_00512 |
| END23_00318 | END23_00535 | END23_00595 | END23_00517 |
| END23_00320 | END23_00551 | END23_00602 | END23_00519 |
| END23_00325 | END23_00569 | END23_00644 | END23_00528 |
| END23_00330 | END23_00573 | END23_00663 | END23_00529 |
| END23_00336 | END23_00574 | END23_00681 | END23_00535 |
| END23_00337 | END23_00575 | END23_00687 | END23_00551 |
| END23_00360 | END23_00578 | END23_00690 | END23_00569 |
| END23_00370 | END23_00580 | END23_00729 | END23_00573 |
| END23_00371 | END23_00595 | END23_00860 | END23_00574 |
| END23_00374 | END23_00602 | END23_00862 | END23_00575 |
| END23_00385 | END23_00644 | END23_00872 | END23_00578 |
| END23_00394 | END23_00663 | END23_00873 | END23_00580 |
| END23_00396 | END23_00681 | END23_00895 | END23_00595 |
| END23_00397 | END23_00687 | END23_00908 | END23_00602 |
| END23_00398 | END23_00690 | END23_00925 | END23_00644 |
| END23_00401 | END23_00729 | END23_00934 | END23_00663 |
| END23_00404 | END23_00856 | END23_00966 | END23_00681 |
| END23_00405 | END23_00859 | END23_00993 | END23_00687 |
| END23_00411 | END23_00860 | END23_01038 | END23_00690 |
| END23_00412 | END23_00862 | END23_01051 | END23_00729 |
| END23_00413 | END23_00872 | END23_01063 | END23_00856 |
| END23_00414 | END23_00873 | END23_01065 | END23_00859 |
| END23_00415 | END23_00895 | END23_01070 | END23_00860 |
| END23_00416 | END23_00908 | END23_01081 | END23_00862 |
| END23_00438 | END23_00925 | END23_01084 | END23_00872 |
| END23_00439 | END23_00934 | END23_01095 | END23_00873 |
| END23_00452 | END23_00966 | END23_01096 | END23_00895 |
| END23_00459 | END23_00993 | END23_01102 | END23_00908 |
| END23_00462 | END23_01019 | END23_01106 | END23_00925 |
| END23_00463 | END23_01038 | END23_01107 | END23_00934 |
| END23_00464 | END23_01051 | END23_01125 | END23_00966 |
| END23_00472 | END23_01063 | END23_01156 | END23_00993 |
| END23_00479 | END23_01065 | END23_01158 | END23_01019 |
| END23_00494 | END23_01070 | END23_01161 | END23_01038 |
| END23_00497 | END23_01081 | END23_01162 | END23_01051 |
| END23_00500 | END23_01084 | END23_01168 | END23_01063 |
| END23_00512 | END23_01095 | END23_01169 | END23_01065 |
| END23_00517 | END23_01096 | END23_01182 | END23_01070 |
| END23_00519 | END23_01102 | END23_01211 | END23_01081 |
| END23_00528 | END23_01106 | END23_01212 | END23_01084 |
| END23_00535 | END23_01107 | END23_01226 | END23_01095 |
| END23_00548 | END23_01125 | END23_01238 | END23_01096 |
| END23_00551 | END23_01156 | END23_01244 | END23_01102 |
| END23_00569 | END23_01158 | END23_01245 | END23_01106 |
| END23_00573 | END23_01161 | END23_01251 | END23_01107 |
| END23_00574 | END23_01162 | END23_01268 | END23_01125 |
| END23_00575 | END23_01168 | END23_01273 | END23_01156 |
| END23_00578 | END23_01169 | END23_01274 | END23_01158 |
| END23_00580 | END23_01182 | END23_01276 | END23_01161 |
| END23_00595 | END23_01211 | END23_01277 | END23_01162 |
| END23_00598 | END23_01212 | END23_01279 | END23_01168 |
| END23_00602 | END23_01226 | END23_01280 | END23_01169 |
| END23_00634 | END23_01238 | END23_01283 | END23_01182 |
| END23_00639 | END23_01244 | END23_01288 | END23_01211 |
| END23_00644 | END23_01245 | END23_01305 | END23_01212 |
| END23_00663 | END23_01251 | END23_01308 | END23_01226 |
| END23_00672 | END23_01254 | END23_01344 | END23_01238 |
| END23_00681 | END23_01259 | END23_01349 | END23_01244 |
| END23_00687 | END23_01265 | END23_01369 | END23_01245 |
| END23_00690 | END23_01268 | END23_01371 | END23_01251 |
| END23_00691 | END23_01269 | END23_01373 | END23_01254 |
| END23_00694 | END23_01273 | END23_01375 | END23_01259 |
| END23_00695 | END23_01274 | END23_01381 | END23_01265 |
| END23_00696 | END23_01276 | END23_01406 | END23_01268 |
| END23_00697 | END23_01277 | END23_01411 | END23_01269 |
| END23_00722 | END23_01279 | END23_01414 | END23_01273 |
| END23_00729 | END23_01280 | END23_01426 | END23_01274 |
| END23_00735 | END23_01283 | END23_01449 | END23_01276 |
| END23_00767 | END23_01288 | END23_01452 | END23_01277 |
| END23_00769 | END23_01305 | END23_01453 | END23_01279 |
| END23_00860 | END23_01308 | END23_01472 | END23_01280 |
| END23_00862 | END23_01320 | END23_01473 | END23_01283 |
| END23_00872 | END23_01344 | END23_01474 | END23_01288 |
| END23_00873 | END23_01349 | END23_01476 | END23_01305 |
| END23_00874 | END23_01369 | END23_01477 | END23_01308 |
| END23_00890 | END23_01371 | END23_01490 | END23_01344 |
| END23_00895 | END23_01373 | END23_01504 | END23_01349 |
| END23_00904 | END23_01375 | END23_01527 | END23_01369 |
| END23_00908 | END23_01381 | END23_01545 | END23_01371 |
| END23_00918 | END23_01406 | END23_01557 | END23_01373 |
| END23_00920 | END23_01407 | END23_01566 | END23_01375 |
| END23_00925 | END23_01410 | END23_01568 | END23_01381 |
| END23_00931 | END23_01411 | END23_01570 | END23_01406 |
| END23_00932 | END23_01414 | END23_01575 | END23_01407 |
| END23_00934 | END23_01426 | END23_01580 | END23_01410 |
| END23_00951 | END23_01449 | END23_01582 | END23_01411 |
| END23_00966 | END23_01452 | END23_01584 | END23_01414 |
| END23_00968 | END23_01453 | END23_01585 | END23_01426 |
| END23_00971 | END23_01472 | END23_01589 | END23_01449 |
| END23_00973 | END23_01473 | END23_01591 | END23_01452 |
| END23_00977 | END23_01474 | END23_01593 | END23_01453 |
| END23_00993 | END23_01476 | END23_01594 | END23_01472 |
| END23_01001 | END23_01477 | END23_01595 | END23_01473 |
| END23_01005 | END23_01490 | END23_01599 | END23_01474 |
| END23_01021 | END23_01504 | END23_01612 | END23_01476 |
| END23_01038 | END23_01527 | END23_01621 | END23_01477 |
| END23_01047 | END23_01545 | END23_01625 | END23_01490 |
| END23_01049 | END23_01557 | END23_01627 | END23_01504 |
| END23_01050 | END23_01566 | END23_01632 | END23_01527 |
| END23_01051 | END23_01568 | END23_01666 | END23_01545 |
| END23_01063 | END23_01570 | END23_01709 | END23_01557 |
| END23_01065 | END23_01575 | END23_01715 | END23_01566 |
| END23_01066 | END23_01580 | END23_01716 | END23_01568 |
| END23_01070 | END23_01582 | END23_01722 | END23_01570 |
| END23_01080 | END23_01584 | END23_01723 | END23_01575 |
| END23_01081 | END23_01585 | END23_01747 | END23_01580 |
| END23_01083 | END23_01589 | END23_01749 | END23_01582 |
| END23_01084 | END23_01591 | END23_01752 | END23_01584 |
| END23_01094 | END23_01593 | END23_01759 | END23_01585 |
| END23_01095 | END23_01594 | END23_01765 | END23_01589 |
| END23_01096 | END23_01595 | END23_01797 | END23_01591 |
| END23_01100 | END23_01599 | END23_01801 | END23_01593 |
| END23_01101 | END23_01612 | END23_01804 | END23_01594 |
| END23_01102 | END23_01621 | END23_01808 | END23_01595 |
| END23_01106 | END23_01625 | END23_01818 | END23_01599 |
| END23_01107 | END23_01627 | END23_01835 | END23_01612 |
| END23_01109 | END23_01632 | END23_01836 | END23_01621 |
| END23_01118 | END23_01666 | END23_01838 | END23_01625 |
| END23_01119 | END23_01709 | END23_01839 | END23_01627 |
| END23_01122 | END23_01715 | END23_01843 | END23_01632 |
| END23_01125 | END23_01716 | END23_01844 | END23_01666 |
| END23_01134 | END23_01722 | END23_01847 | END23_01709 |
| END23_01141 | END23_01723 | END23_01850 | END23_01715 |
| END23_01150 | END23_01747 | END23_01852 | END23_01716 |
| END23_01156 | END23_01749 | END23_01854 | END23_01722 |
| END23_01158 | END23_01752 | END23_01858 | END23_01723 |
| END23_01159 | END23_01759 | END23_01860 | END23_01747 |
| END23_01161 | END23_01765 | END23_01865 | END23_01749 |
| END23_01162 | END23_01797 | END23_01870 | END23_01752 |
| END23_01165 | END23_01801 | END23_01871 | END23_01759 |
| END23_01166 | END23_01804 | END23_01872 | END23_01765 |
| END23_01167 | END23_01808 | END23_01873 | END23_01797 |
| END23_01168 | END23_01818 | END23_01878 | END23_01801 |
| END23_01169 | END23_01835 | END23_01885 | END23_01804 |
| END23_01170 | END23_01836 | END23_01906 | END23_01808 |
| END23_01173 | END23_01838 | END23_01930 | END23_01818 |
| END23_01182 | END23_01839 | END23_01932 | END23_01835 |
| END23_01184 | END23_01843 | END23_01939 | END23_01836 |
| END23_01189 | END23_01844 | END23_01943 | END23_01838 |
| END23_01190 | END23_01847 | END23_01972 | END23_01839 |
| END23_01191 | END23_01850 | END23_01988 | END23_01843 |
| END23_01192 | END23_01852 | END23_01989 | END23_01844 |
| END23_01211 | END23_01854 | END23_01990 | END23_01847 |
| END23_01212 | END23_01858 | END23_01992 | END23_01850 |
| END23_01226 | END23_01860 | END23_01993 | END23_01852 |
| END23_01229 | END23_01865 | END23_01995 | END23_01854 |
| END23_01235 | END23_01870 | END23_01997 | END23_01858 |
| END23_01238 | END23_01871 | END23_01998 | END23_01860 |
| END23_01244 | END23_01872 | END23_02005 | END23_01865 |
| END23_01245 | END23_01873 | END23_02009 | END23_01870 |
| END23_01251 | END23_01878 | END23_02014 | END23_01871 |
| END23_01264 | END23_01884 | END23_02028 | END23_01872 |
| END23_01268 | END23_01885 | END23_02058 | END23_01873 |
| END23_01270 | END23_01906 | END23_02073 | END23_01878 |
| END23_01273 | END23_01930 | END23_02087 | END23_01884 |
| END23_01274 | END23_01932 | END23_02098 | END23_01885 |
| END23_01276 | END23_01939 | END23_02119 | END23_01906 |
| END23_01277 | END23_01943 | END23_02121 | END23_01930 |
| END23_01278 | END23_01968 | END23_02123 | END23_01932 |
| END23_01279 | END23_01972 | END23_02125 | END23_01939 |
| END23_01280 | END23_01988 | END23_02130 | END23_01943 |
| END23_01283 | END23_01989 | END23_02140 | END23_01968 |
| END23_01288 | END23_01990 | END23_02146 | END23_01972 |
| END23_01303 | END23_01992 | END23_02166 | END23_01988 |
| END23_01305 | END23_01993 | END23_02185 | END23_01989 |
| END23_01306 | END23_01994 | END23_02186 | END23_01990 |
| END23_01308 | END23_01995 | END23_02195 | END23_01992 |
| END23_01310 | END23_01997 | END23_02199 | END23_01993 |
| END23_01311 | END23_01998 | END23_02237 | END23_01994 |
| END23_01312 | END23_01999 | END23_02242 | END23_01995 |
| END23_01314 | END23_02000 | END23_02289 | END23_01997 |
| END23_01317 | END23_02003 | END23_02311 | END23_01998 |
| END23_01321 | END23_02004 | END23_02313 | END23_01999 |
| END23_01322 | END23_02005 | END23_02314 | END23_02000 |
| END23_01323 | END23_02009 | END23_02315 | END23_02003 |
| END23_01326 | END23_02014 | END23_02316 | END23_02004 |
| END23_01338 | END23_02028 | END23_02318 | END23_02005 |
| END23_01340 | END23_02058 | END23_02331 | END23_02009 |
| END23_01342 | END23_02073 | END23_02359 | END23_02014 |
| END23_01343 | END23_02087 | END23_02361 | END23_02028 |
| END23_01344 | END23_02093 | END23_02362 | END23_02058 |
| END23_01349 | END23_02098 | END23_02363 | END23_02073 |
| END23_01353 | END23_02119 | END23_02366 | END23_02087 |
| END23_01369 | END23_02121 | END23_02384 | END23_02093 |
| END23_01371 | END23_02123 | END23_02397 | END23_02098 |
| END23_01373 | END23_02125 | END23_02405 | END23_02119 |
| END23_01375 | END23_02130 | END23_02434 | END23_02121 |
| END23_01378 | END23_02140 | END23_02453 | END23_02123 |
| END23_01381 | END23_02146 | END23_02508 | END23_02125 |
| END23_01382 | END23_02166 | END23_02509 | END23_02130 |
| END23_01389 | END23_02185 | END23_02510 | END23_02140 |
| END23_01393 | END23_02186 | END23_02511 | END23_02146 |
| END23_01404 | END23_02195 | END23_02512 | END23_02166 |
| END23_01406 | END23_02199 | END23_02518 | END23_02185 |
| END23_01411 | END23_02237 | END23_02522 | END23_02186 |
| END23_01412 | END23_02242 | END23_02530 | END23_02195 |
| END23_01414 | END23_02248 | END23_02532 | END23_02199 |
| END23_01426 | END23_02289 | END23_02533 | END23_02237 |
| END23_01436 | END23_02311 | END23_02547 | END23_02242 |
| END23_01449 | END23_02313 | END23_02560 | END23_02248 |
| END23_01450 | END23_02314 | END23_02570 | END23_02289 |
| END23_01452 | END23_02315 | END23_02589 | END23_02311 |
| END23_01453 | END23_02316 | END23_02590 | END23_02313 |
| END23_01472 | END23_02318 | END23_02591 | END23_02314 |
| END23_01473 | END23_02331 | END23_02599 | END23_02315 |
| END23_01474 | END23_02358 | END23_02602 | END23_02316 |
| END23_01475 | END23_02359 | END23_02606 | END23_02318 |
| END23_01476 | END23_02361 | END23_02626 | END23_02331 |
| END23_01477 | END23_02362 | END23_02668 | END23_02358 |
| END23_01478 | END23_02363 | END23_02676 | END23_02359 |
| END23_01480 | END23_02366 | END23_02690 | END23_02361 |
| END23_01490 | END23_02384 | END23_02695 | END23_02362 |
| END23_01493 | END23_02397 | END23_02696 | END23_02363 |
| END23_01494 | END23_02405 | END23_02697 | END23_02366 |
| END23_01495 | END23_02434 | END23_02698 | END23_02384 |
| END23_01499 | END23_02453 | END23_02699 | END23_02397 |
| END23_01502 | END23_02508 | END23_02703 | END23_02405 |
| END23_01504 | END23_02509 | END23_02704 | END23_02434 |
| END23_01527 | END23_02510 | END23_02706 | END23_02453 |
| END23_01545 | END23_02511 | END23_02716 | END23_02508 |
| END23_01554 | END23_02512 | END23_02723 | END23_02509 |
| END23_01556 | END23_02518 | END23_02724 | END23_02510 |
| END23_01557 | END23_02522 | END23_02727 | END23_02511 |
| END23_01566 | END23_02530 | END23_02728 | END23_02512 |
| END23_01568 | END23_02532 | END23_02743 | END23_02518 |
| END23_01570 | END23_02533 | END23_02746 | END23_02522 |
| END23_01571 | END23_02547 | END23_02748 | END23_02530 |
| END23_01575 | END23_02560 | END23_02764 | END23_02532 |
| END23_01580 | END23_02563 | END23_02767 | END23_02533 |
| END23_01582 | END23_02570 | END23_02783 | END23_02547 |
| END23_01583 | END23_02589 | END23_02786 | END23_02560 |
| END23_01584 | END23_02590 | END23_02791 | END23_02570 |
| END23_01585 | END23_02591 | END23_02795 | END23_02589 |
| END23_01589 | END23_02599 | END23_02800 | END23_02590 |
| END23_01590 | END23_02602 | END23_02803 | END23_02591 |
| END23_01591 | END23_02606 | END23_02811 | END23_02599 |
| END23_01593 | END23_02615 | END23_02812 | END23_02602 |
| END23_01594 | END23_02616 | END23_02815 | END23_02606 |
| END23_01595 | END23_02626 | END23_02853 | END23_02615 |
| END23_01597 | END23_02668 | END23_02856 | END23_02616 |
| END23_01599 | END23_02676 | END23_02858 | END23_02626 |
| END23_01602 | END23_02690 | END23_02860 | END23_02668 |
| END23_01610 | END23_02695 | END23_02877 | END23_02676 |
| END23_01612 | END23_02696 | END23_02878 | END23_02690 |
| END23_01621 | END23_02697 | END23_02880 | END23_02695 |
| END23_01623 | END23_02698 | END23_02886 | END23_02696 |
| END23_01625 | END23_02699 | END23_02922 | END23_02697 |
| END23_01627 | END23_02703 | END23_02929 | END23_02698 |
| END23_01628 | END23_02704 | END23_02950 | END23_02699 |
| END23_01632 | END23_02706 | END23_02952 | END23_02703 |
| END23_01666 | END23_02716 | END23_02954 | END23_02704 |
| END23_01690 | END23_02723 | END23_02962 | END23_02706 |
| END23_01709 | END23_02724 | END23_02963 | END23_02716 |
| END23_01715 | END23_02727 | END23_02964 | END23_02723 |
| END23_01716 | END23_02728 | END23_02965 | END23_02724 |
| END23_01722 | END23_02743 | END23_02966 | END23_02727 |
| END23_01723 | END23_02746 | END23_02968 | END23_02728 |
| END23_01734 | END23_02748 | END23_02969 | END23_02743 |
| END23_01747 | END23_02764 | END23_02972 | END23_02746 |
| END23_01749 | END23_02767 | END23_02999 | END23_02748 |
| END23_01751 | END23_02769 | END23_03005 | END23_02764 |
| END23_01752 | END23_02772 | END23_03013 | END23_02767 |
| END23_01759 | END23_02783 | END23_03014 | END23_02769 |
| END23_01765 | END23_02786 | END23_03015 | END23_02772 |
| END23_01769 | END23_02791 | END23_03020 | END23_02783 |
| END23_01781 | END23_02795 | END23_03025 | END23_02786 |
| END23_01791 | END23_02800 | END23_03040 | END23_02791 |
| END23_01797 | END23_02803 | END23_03045 | END23_02795 |
| END23_01801 | END23_02811 | END23_03047 | END23_02800 |
| END23_01804 | END23_02812 | END23_03048 | END23_02803 |
| END23_01808 | END23_02815 | END23_03049 | END23_02811 |
| END23_01818 | END23_02853 | END23_03050 | END23_02812 |
| END23_01831 | END23_02856 | END23_03051 | END23_02815 |
| END23_01835 | END23_02858 | END23_03052 | END23_02853 |
| END23_01836 | END23_02860 | END23_03053 | END23_02856 |
| END23_01838 | END23_02877 | END23_03086 | END23_02858 |
| END23_01839 | END23_02878 | END23_03087 | END23_02860 |
| END23_01840 | END23_02880 | END23_03125 | END23_02877 |
| END23_01841 | END23_02886 | END23_03129 | END23_02878 |
| END23_01842 | END23_02922 | END23_03130 | END23_02880 |
| END23_01843 | END23_02929 | END23_03139 | END23_02886 |
| END23_01844 | END23_02950 | END23_03140 | END23_02922 |
| END23_01845 | END23_02952 | END23_03170 | END23_02929 |
| END23_01847 | END23_02954 | END23_03179 | END23_02950 |
| END23_01848 | END23_02962 | END23_03240 | END23_02952 |
| END23_01850 | END23_02963 | END23_03261 | END23_02954 |
| END23_01851 | END23_02964 | END23_03284 | END23_02962 |
| END23_01852 | END23_02965 | END23_03287 | END23_02963 |
| END23_01853 | END23_02966 | END23_03291 | END23_02964 |
| END23_01854 | END23_02968 | END23_03292 | END23_02965 |
| END23_01857 | END23_02969 | END23_03296 | END23_02966 |
| END23_01858 | END23_02972 | END23_03303 | END23_02968 |
| END23_01859 | END23_02999 | END23_03312 | END23_02969 |
| END23_01860 | END23_03005 | END23_03314 | END23_02972 |
| END23_01861 | END23_03013 | END23_03318 | END23_02999 |
| END23_01862 | END23_03014 | END23_03344 | END23_03005 |
| END23_01863 | END23_03015 | END23_03359 | END23_03013 |
| END23_01864 | END23_03020 | END23_03398 | END23_03014 |
| END23_01865 | END23_03025 | END23_03406 | END23_03015 |
| END23_01869 | END23_03040 | END23_03410 | END23_03020 |
| END23_01870 | END23_03042 | END23_03411 | END23_03025 |
| END23_01871 | END23_03045 | END23_03412 | END23_03040 |
| END23_01872 | END23_03047 | END23_03413 | END23_03042 |
| END23_01873 | END23_03048 | END23_03414 | END23_03045 |
| END23_01878 | END23_03049 | END23_03415 | END23_03047 |
| END23_01879 | END23_03050 | END23_03416 | END23_03048 |
| END23_01882 | END23_03051 | END23_03417 | END23_03049 |
| END23_01885 | END23_03052 | END23_03418 | END23_03050 |
| END23_01890 | END23_03053 | END23_03419 | END23_03051 |
| END23_01895 | END23_03086 | END23_03420 | END23_03052 |
| END23_01906 | END23_03087 | END23_03421 | END23_03053 |
| END23_01930 | END23_03125 | END23_03422 | END23_03086 |
| END23_01931 | END23_03129 | END23_03423 | END23_03087 |
| END23_01932 | END23_03130 | END23_03450 | END23_03125 |
| END23_01939 | END23_03139 | END23_03490 | END23_03129 |
| END23_01943 | END23_03140 | END23_03496 | END23_03130 |
| END23_01972 | END23_03170 | END23_03500 | END23_03139 |
| END23_01974 | END23_03179 | END23_03506 | END23_03140 |
| END23_01988 | END23_03240 | END23_03529 | END23_03170 |
| END23_01989 | END23_03261 | END23_03543 | END23_03179 |
| END23_01990 | END23_03274 | END23_03544 | END23_03234 |
| END23_01992 | END23_03284 | END23_03549 | END23_03240 |
| END23_01993 | END23_03287 | END23_03562 | END23_03261 |
| END23_01995 | END23_03291 | END23_03586 | END23_03274 |
| END23_01997 | END23_03292 | END23_03613 | END23_03284 |
| END23_01998 | END23_03296 | END23_03636 | END23_03287 |
| END23_02005 | END23_03303 | END23_03645 | END23_03291 |
| END23_02007 | END23_03306 | END23_03653 | END23_03292 |
| END23_02008 | END23_03312 | END23_03654 | END23_03296 |
| END23_02009 | END23_03314 | END23_03664 | END23_03303 |
| END23_02010 | END23_03318 | END23_03673 | END23_03306 |
| END23_02011 | END23_03344 | END23_03674 | END23_03312 |
| END23_02014 | END23_03359 | END23_03681 | END23_03314 |
| END23_02028 | END23_03398 | END23_03682 | END23_03318 |
| END23_02041 | END23_03406 | END23_03735 | END23_03344 |
| END23_02053 | END23_03410 | END23_03746 | END23_03359 |
| END23_02058 | END23_03411 | END23_03777 | END23_03398 |
| END23_02068 | END23_03412 | END23_03783 | END23_03406 |
| END23_02073 | END23_03413 | END23_03851 | END23_03410 |
| END23_02087 | END23_03414 | END23_03859 | END23_03411 |
| END23_02098 | END23_03415 | END23_03868 | END23_03412 |
| END23_02113 | END23_03416 | END23_03869 | END23_03413 |
| END23_02119 | END23_03417 | END23_03889 | END23_03414 |
| END23_02121 | END23_03418 | END23_03898 | END23_03415 |
| END23_02122 | END23_03419 | END23_03903 | END23_03416 |
| END23_02123 | END23_03420 | END23_03932 | END23_03417 |
| END23_02124 | END23_03421 | END23_03933 | END23_03418 |
| END23_02125 | END23_03422 | END23_03935 | END23_03419 |
| END23_02130 | END23_03423 | END23_03938 | END23_03420 |
| END23_02138 | END23_03450 | END23_03942 | END23_03421 |
| END23_02140 | END23_03490 | END23_03943 | END23_03422 |
| END23_02143 | END23_03496 | END23_03944 | END23_03423 |
| END23_02146 | END23_03500 | END23_03949 | END23_03450 |
| END23_02163 | END23_03506 | END23_03950 | END23_03490 |
| END23_02166 | END23_03512 | END23_03965 | END23_03496 |
| END23_02175 | END23_03529 | END23_03971 | END23_03500 |
| END23_02183 | END23_03543 | END23_03973 | END23_03506 |
| END23_02185 | END23_03544 | END23_03974 | END23_03512 |
| END23_02186 | END23_03549 | END23_03975 | END23_03529 |
| END23_02188 | END23_03558 | END23_03976 | END23_03543 |
| END23_02192 | END23_03562 | END23_03977 | END23_03544 |
| END23_02195 | END23_03586 | END23_03986 | END23_03549 |
| END23_02199 | END23_03613 | END23_04002 | END23_03558 |
| END23_02209 | END23_03636 | END23_04052 | END23_03562 |
| END23_02232 | END23_03645 | END23_04057 | END23_03586 |
| END23_02237 | END23_03653 | END23_04064 | END23_03613 |
| END23_02242 | END23_03654 | END23_04081 | END23_03636 |
| END23_02244 | END23_03664 | END23_04212 | END23_03645 |
| END23_02269 | END23_03670 | END23_04219 | END23_03653 |
| END23_02289 | END23_03673 | END23_04221 | END23_03654 |
| END23_02298 | END23_03674 | END23_04222 | END23_03664 |
| END23_02309 | END23_03681 | END23_04223 | END23_03670 |
| END23_02311 | END23_03682 | END23_04224 | END23_03673 |
| END23_02312 | END23_03735 | END23_04226 | END23_03674 |
| END23_02313 | END23_03746 | END23_04248 | END23_03681 |
| END23_02314 | END23_03777 | END23_04249 | END23_03682 |
| END23_02315 | END23_03783 | END23_04269 | END23_03735 |
| END23_02316 | END23_03784 | END23_04285 | END23_03746 |
| END23_02317 | END23_03851 | END23_04287 | END23_03777 |
| END23_02318 | END23_03859 | END23_04289 | END23_03783 |
| END23_02321 | END23_03868 | END23_04290 | END23_03784 |
| END23_02322 | END23_03869 | END23_04291 | END23_03851 |
| END23_02329 | END23_03889 | END23_04292 | END23_03859 |
| END23_02330 | END23_03897 | END23_04293 | END23_03868 |
| END23_02331 | END23_03898 | END23_04296 | END23_03869 |
| END23_02332 | END23_03903 |  | END23_03889 |
| END23_02337 | END23_03932 |  | END23_03897 |
| END23_02338 | END23_03933 |  | END23_03898 |
| END23_02339 | END23_03935 |  | END23_03903 |
| END23_02340 | END23_03938 |  | END23_03932 |
| END23_02345 | END23_03942 |  | END23_03933 |
| END23_02348 | END23_03943 |  | END23_03935 |
| END23_02349 | END23_03944 |  | END23_03938 |
| END23_02350 | END23_03949 |  | END23_03942 |
| END23_02355 | END23_03950 |  | END23_03943 |
| END23_02359 | END23_03965 |  | END23_03944 |
| END23_02361 | END23_03971 |  | END23_03949 |
| END23_02362 | END23_03973 |  | END23_03950 |
| END23_02363 | END23_03974 |  | END23_03965 |
| END23_02365 | END23_03975 |  | END23_03971 |
| END23_02366 | END23_03976 |  | END23_03973 |
| END23_02384 | END23_03977 |  | END23_03974 |
| END23_02397 | END23_03986 |  | END23_03975 |
| END23_02404 | END23_04002 |  | END23_03976 |
| END23_02405 | END23_04004 |  | END23_03977 |
| END23_02434 | END23_04052 |  | END23_03986 |
| END23_02453 | END23_04057 |  | END23_04002 |
| END23_02492 | END23_04064 |  | END23_04004 |
| END23_02493 | END23_04081 |  | END23_04052 |
| END23_02494 | END23_04212 |  | END23_04057 |
| END23_02503 | END23_04219 |  | END23_04064 |
| END23_02507 | END23_04221 |  | END23_04081 |
| END23_02508 | END23_04222 |  | END23_04212 |
| END23_02509 | END23_04223 |  | END23_04219 |
| END23_02510 | END23_04224 |  | END23_04221 |
| END23_02511 | END23_04226 |  | END23_04222 |
| END23_02512 | END23_04248 |  | END23_04223 |
| END23_02516 | END23_04249 |  | END23_04224 |
| END23_02518 | END23_04269 |  | END23_04226 |
| END23_02522 | END23_04285 |  | END23_04248 |
| END23_02523 | END23_04287 |  | END23_04249 |
| END23_02524 | END23_04289 |  | END23_04269 |
| END23_02530 | END23_04290 |  | END23_04285 |
| END23_02532 | END23_04291 |  | END23_04287 |
| END23_02533 | END23_04292 |  | END23_04289 |
| END23_02547 | END23_04293 |  | END23_04290 |
| END23_02555 | END23_04296 |  | END23_04291 |
| END23_02559 |  |  | END23_04292 |
| END23_02560 |  |  | END23_04293 |
| END23_02570 |  |  | END23_04296 |
| END23_02571 |  |  |  |
| END23_02573 |  |  |  |
| END23_02584 |  |  |  |
| END23_02589 |  |  |  |
| END23_02590 |  |  |  |
| END23_02591 |  |  |  |
| END23_02599 |  |  |  |
| END23_02600 |  |  |  |
| END23_02602 |  |  |  |
| END23_02606 |  |  |  |
| END23_02609 |  |  |  |
| END23_02617 |  |  |  |
| END23_02626 |  |  |  |
| END23_02643 |  |  |  |
| END23_02647 |  |  |  |
| END23_02661 |  |  |  |
| END23_02667 |  |  |  |
| END23_02668 |  |  |  |
| END23_02676 |  |  |  |
| END23_02680 |  |  |  |
| END23_02681 |  |  |  |
| END23_02682 |  |  |  |
| END23_02690 |  |  |  |
| END23_02695 |  |  |  |
| END23_02696 |  |  |  |
| END23_02697 |  |  |  |
| END23_02698 |  |  |  |
| END23_02699 |  |  |  |
| END23_02702 |  |  |  |
| END23_02703 |  |  |  |
| END23_02704 |  |  |  |
| END23_02706 |  |  |  |
| END23_02708 |  |  |  |
| END23_02716 |  |  |  |
| END23_02723 |  |  |  |
| END23_02724 |  |  |  |
| END23_02727 |  |  |  |
| END23_02728 |  |  |  |
| END23_02741 |  |  |  |
| END23_02742 |  |  |  |
| END23_02743 |  |  |  |
| END23_02746 |  |  |  |
| END23_02748 |  |  |  |
| END23_02764 |  |  |  |
| END23_02767 |  |  |  |
| END23_02770 |  |  |  |
| END23_02775 |  |  |  |
| END23_02776 |  |  |  |
| END23_02777 |  |  |  |
| END23_02783 |  |  |  |
| END23_02786 |  |  |  |
| END23_02791 |  |  |  |
| END23_02795 |  |  |  |
| END23_02800 |  |  |  |
| END23_02803 |  |  |  |
| END23_02807 |  |  |  |
| END23_02811 |  |  |  |
| END23_02812 |  |  |  |
| END23_02815 |  |  |  |
| END23_02853 |  |  |  |
| END23_02856 |  |  |  |
| END23_02858 |  |  |  |
| END23_02860 |  |  |  |
| END23_02869 |  |  |  |
| END23_02870 |  |  |  |
| END23_02877 |  |  |  |
| END23_02878 |  |  |  |
| END23_02880 |  |  |  |
| END23_02886 |  |  |  |
| END23_02922 |  |  |  |
| END23_02928 |  |  |  |
| END23_02929 |  |  |  |
| END23_02943 |  |  |  |
| END23_02949 |  |  |  |
| END23_02950 |  |  |  |
| END23_02951 |  |  |  |
| END23_02952 |  |  |  |
| END23_02954 |  |  |  |
| END23_02958 |  |  |  |
| END23_02959 |  |  |  |
| END23_02960 |  |  |  |
| END23_02962 |  |  |  |
| END23_02963 |  |  |  |
| END23_02964 |  |  |  |
| END23_02965 |  |  |  |
| END23_02966 |  |  |  |
| END23_02967 |  |  |  |
| END23_02968 |  |  |  |
| END23_02969 |  |  |  |
| END23_02971 |  |  |  |
| END23_02972 |  |  |  |
| END23_02977 |  |  |  |
| END23_02989 |  |  |  |
| END23_02995 |  |  |  |
| END23_02996 |  |  |  |
| END23_02998 |  |  |  |
| END23_02999 |  |  |  |
| END23_03003 |  |  |  |
| END23_03005 |  |  |  |
| END23_03013 |  |  |  |
| END23_03014 |  |  |  |
| END23_03015 |  |  |  |
| END23_03017 |  |  |  |
| END23_03019 |  |  |  |
| END23_03020 |  |  |  |
| END23_03024 |  |  |  |
| END23_03025 |  |  |  |
| END23_03026 |  |  |  |
| END23_03030 |  |  |  |
| END23_03040 |  |  |  |
| END23_03045 |  |  |  |
| END23_03046 |  |  |  |
| END23_03047 |  |  |  |
| END23_03048 |  |  |  |
| END23_03049 |  |  |  |
| END23_03050 |  |  |  |
| END23_03051 |  |  |  |
| END23_03052 |  |  |  |
| END23_03053 |  |  |  |
| END23_03061 |  |  |  |
| END23_03062 |  |  |  |
| END23_03063 |  |  |  |
| END23_03064 |  |  |  |
| END23_03066 |  |  |  |
| END23_03086 |  |  |  |
| END23_03087 |  |  |  |
| END23_03120 |  |  |  |
| END23_03121 |  |  |  |
| END23_03124 |  |  |  |
| END23_03125 |  |  |  |
| END23_03126 |  |  |  |
| END23_03129 |  |  |  |
| END23_03130 |  |  |  |
| END23_03131 |  |  |  |
| END23_03132 |  |  |  |
| END23_03133 |  |  |  |
| END23_03134 |  |  |  |
| END23_03135 |  |  |  |
| END23_03139 |  |  |  |
| END23_03140 |  |  |  |
| END23_03141 |  |  |  |
| END23_03143 |  |  |  |
| END23_03149 |  |  |  |
| END23_03157 |  |  |  |
| END23_03170 |  |  |  |
| END23_03171 |  |  |  |
| END23_03174 |  |  |  |
| END23_03179 |  |  |  |
| END23_03189 |  |  |  |
| END23_03192 |  |  |  |
| END23_03198 |  |  |  |
| END23_03203 |  |  |  |
| END23_03223 |  |  |  |
| END23_03234 |  |  |  |
| END23_03235 |  |  |  |
| END23_03240 |  |  |  |
| END23_03241 |  |  |  |
| END23_03261 |  |  |  |
| END23_03264 |  |  |  |
| END23_03268 |  |  |  |
| END23_03284 |  |  |  |
| END23_03287 |  |  |  |
| END23_03291 |  |  |  |
| END23_03292 |  |  |  |
| END23_03296 |  |  |  |
| END23_03303 |  |  |  |
| END23_03304 |  |  |  |
| END23_03305 |  |  |  |
| END23_03312 |  |  |  |
| END23_03313 |  |  |  |
| END23_03314 |  |  |  |
| END23_03318 |  |  |  |
| END23_03325 |  |  |  |
| END23_03344 |  |  |  |
| END23_03359 |  |  |  |
| END23_03389 |  |  |  |
| END23_03390 |  |  |  |
| END23_03398 |  |  |  |
| END23_03406 |  |  |  |
| END23_03410 |  |  |  |
| END23_03411 |  |  |  |
| END23_03412 |  |  |  |
| END23_03413 |  |  |  |
| END23_03414 |  |  |  |
| END23_03415 |  |  |  |
| END23_03416 |  |  |  |
| END23_03417 |  |  |  |
| END23_03418 |  |  |  |
| END23_03419 |  |  |  |
| END23_03420 |  |  |  |
| END23_03421 |  |  |  |
| END23_03422 |  |  |  |
| END23_03423 |  |  |  |
| END23_03426 |  |  |  |
| END23_03431 |  |  |  |
| END23_03435 |  |  |  |
| END23_03436 |  |  |  |
| END23_03446 |  |  |  |
| END23_03447 |  |  |  |
| END23_03450 |  |  |  |
| END23_03483 |  |  |  |
| END23_03490 |  |  |  |
| END23_03491 |  |  |  |
| END23_03496 |  |  |  |
| END23_03497 |  |  |  |
| END23_03500 |  |  |  |
| END23_03502 |  |  |  |
| END23_03506 |  |  |  |
| END23_03515 |  |  |  |
| END23_03529 |  |  |  |
| END23_03543 |  |  |  |
| END23_03544 |  |  |  |
| END23_03546 |  |  |  |
| END23_03549 |  |  |  |
| END23_03562 |  |  |  |
| END23_03581 |  |  |  |
| END23_03586 |  |  |  |
| END23_03591 |  |  |  |
| END23_03592 |  |  |  |
| END23_03593 |  |  |  |
| END23_03613 |  |  |  |
| END23_03636 |  |  |  |
| END23_03645 |  |  |  |
| END23_03648 |  |  |  |
| END23_03649 |  |  |  |
| END23_03653 |  |  |  |
| END23_03654 |  |  |  |
| END23_03656 |  |  |  |
| END23_03664 |  |  |  |
| END23_03673 |  |  |  |
| END23_03674 |  |  |  |
| END23_03675 |  |  |  |
| END23_03681 |  |  |  |
| END23_03682 |  |  |  |
| END23_03735 |  |  |  |
| END23_03746 |  |  |  |
| END23_03764 |  |  |  |
| END23_03777 |  |  |  |
| END23_03783 |  |  |  |
| END23_03785 |  |  |  |
| END23_03790 |  |  |  |
| END23_03791 |  |  |  |
| END23_03792 |  |  |  |
| END23_03793 |  |  |  |
| END23_03794 |  |  |  |
| END23_03795 |  |  |  |
| END23_03796 |  |  |  |
| END23_03830 |  |  |  |
| END23_03846 |  |  |  |
| END23_03851 |  |  |  |
| END23_03857 |  |  |  |
| END23_03858 |  |  |  |
| END23_03859 |  |  |  |
| END23_03860 |  |  |  |
| END23_03861 |  |  |  |
| END23_03865 |  |  |  |
| END23_03867 |  |  |  |
| END23_03868 |  |  |  |
| END23_03869 |  |  |  |
| END23_03880 |  |  |  |
| END23_03889 |  |  |  |
| END23_03893 |  |  |  |
| END23_03894 |  |  |  |
| END23_03898 |  |  |  |
| END23_03903 |  |  |  |
| END23_03912 |  |  |  |
| END23_03913 |  |  |  |
| END23_03914 |  |  |  |
| END23_03917 |  |  |  |
| END23_03920 |  |  |  |
| END23_03921 |  |  |  |
| END23_03932 |  |  |  |
| END23_03933 |  |  |  |
| END23_03935 |  |  |  |
| END23_03937 |  |  |  |
| END23_03938 |  |  |  |
| END23_03941 |  |  |  |
| END23_03942 |  |  |  |
| END23_03943 |  |  |  |
| END23_03944 |  |  |  |
| END23_03945 |  |  |  |
| END23_03948 |  |  |  |
| END23_03949 |  |  |  |
| END23_03950 |  |  |  |
| END23_03951 |  |  |  |
| END23_03960 |  |  |  |
| END23_03961 |  |  |  |
| END23_03963 |  |  |  |
| END23_03965 |  |  |  |
| END23_03971 |  |  |  |
| END23_03972 |  |  |  |
| END23_03973 |  |  |  |
| END23_03974 |  |  |  |
| END23_03975 |  |  |  |
| END23_03976 |  |  |  |
| END23_03977 |  |  |  |
| END23_03986 |  |  |  |
| END23_04001 |  |  |  |
| END23_04002 |  |  |  |
| END23_04020 |  |  |  |
| END23_04035 |  |  |  |
| END23_04036 |  |  |  |
| END23_04037 |  |  |  |
| END23_04044 |  |  |  |
| END23_04052 |  |  |  |
| END23_04057 |  |  |  |
| END23_04063 |  |  |  |
| END23_04064 |  |  |  |
| END23_04081 |  |  |  |
| END23_04104 |  |  |  |
| END23_04110 |  |  |  |
| END23_04111 |  |  |  |
| END23_04114 |  |  |  |
| END23_04121 |  |  |  |
| END23_04131 |  |  |  |
| END23_04135 |  |  |  |
| END23_04154 |  |  |  |
| END23_04197 |  |  |  |
| END23_04212 |  |  |  |
| END23_04219 |  |  |  |
| END23_04221 |  |  |  |
| END23_04222 |  |  |  |
| END23_04223 |  |  |  |
| END23_04224 |  |  |  |
| END23_04226 |  |  |  |
| END23_04230 |  |  |  |
| END23_04248 |  |  |  |
| END23_04249 |  |  |  |
| END23_04250 |  |  |  |
| END23_04260 |  |  |  |
| END23_04262 |  |  |  |
| END23_04269 |  |  |  |
| END23_04271 |  |  |  |
| END23_04275 |  |  |  |
| END23_04282 |  |  |  |
| END23_04285 |  |  |  |
| END23_04287 |  |  |  |
| END23_04288 |  |  |  |
| END23_04289 |  |  |  |
| END23_04290 |  |  |  |
| END23_04291 |  |  |  |
| END23_04292 |  |  |  |
| END23_04293 |  |  |  |
| END23_04294 |  |  |  |
| END23_04296 |  |  |  |
