# Supplementary material for: Comparative Genomics Reveals Metabolic Specificity of Endozoicomonas Isolated from a Marine Sponge and the Genomic Repertoire for Host-Bacteria Symbioses
Source: Microorganisms. 2019 Nov 30;7(12):635. doi: 10.3390/microorganisms7120635 (PMC6955870; doi:10.3390/microorganisms7120635)
Supplement: Supplementary file 1 [file microorganisms-07-00635-s001.zip › supplementaryMaterials/TableS3.docx]

**Supplementary Table S3**. List of essential genes predicted within the genome-specific genes of *Endozoicomonas* sp. OPT23

| **Query Protein** | **Function Predicted by PRODIGAL** | **DEG_AC Number** | **Function Predicted by DEG** | **Class** |
| --- | --- | --- | --- | --- |
| END23_00063 | hypothetical protein | DEG10470425 | hypothetical protein | - |
| END23_00078 | hypothetical protein | DEG10180415 | Hypothetical protein ygaP | - |
| END23_00101 | NnrS protein | DEG10350292 | hypothetical protein | - |
| END23_00142 | L-Rhamnulokinase | DEG10070239 | Fucose kinase | Carbohydrate transport and metabolism |
| END23_00131 | Dihydroanticapsin 7-dehydrogenase | DEG10100554 | short chain dehydrogenase | - |
| END23_00213 | Lactate utilization protein B | DEG10470121 | lactate utilization protein B | - |
| END23_00300 | Levanase | DEG10230058 | arabinosidase | - |
| END23_00374 | Inner membrane protein YccF | DEG10340292 | hypothetical protein | - |
| END23_00396 | Periplasmic binding protein | DEG10470170 | ABC transporter permease | - |
| END23_00512 | Amino-acid permease RocC | DEG10470067 | amino acid permease | - |
| END23_00529 | OsmC-like protein | DEG10150226 | hypothetical protein | Adaptation, protection |
| END23_00569 | Cob(I)yrinic acid a,c-diamide adenosyltransferase | DEG10180219 | COB(I)alamin adenosyltransferase (EC 2.5.1.17) | - |
| END23_00573 | Aerobic cobaltochelatase subunit CobN | DEG10400022 | cobaltochelatase subunit CobN | - |
| END23_00574 | MotA/TolQ/ExbB proton channel family protein | DEG10150184 | hypothetical protein | Hypothetical, unclassified, unknown |
| END23_01051 | Bacterial protein of unknown function (YtfJ_HI0045) | DEG10050022 | hypothetical protein | - |
| END23_01106 | Mitochondrial biogenesis AIM24 | DEG10470431 | TIGR00266 family protein | - |
| END23_01125 | Nicotinamide riboside transporter PnuC | DEG10070222 | Conserved hypothetical protein | Function unknown |
| END23_01158 | Glyoxalase/Bleomycin resistance protein/Dioxygenase superfamily protein | DEG10030674 | hypothetical protein | - |
| END23_01169 | Acetyltransferase (GNAT) family protein | DEG10150172 | putative acetyltransferase | Putative enzymes |
| END23_01182 | Invasion gene expression up-regulator, SirB | DEG10030422 | hypothetical protein | - |
| END23_01245 | putative ACR, YggU family | DEG10280308 | hypothetical protein | - |
| END23_01407 | Choline/ethanolamine kinase | DEG10060293 | choline/ethanolamine kinase, putative | Fatty acid and phospholipid metabolism |
| END23_01411 | GDP-L-fucose synthase | DEG10420227 | nucleotide sugar dehydratase | - |
| END23_01453 | NAD dependent epimerase/dehydratase family protein | DEG10280373 | NAD-dependent epimerase/dehydratase | - |
| END23_01599 | Hydantoin racemase | DEG10350244 | amino acid racemase | - |
| END23_01843 | NADP-reducing hydrogenase subunit HndA | DEG10120377 | NADH dehydrogenase I, E subunit | energy metabolism |
| END23_01844 | NADP-reducing hydrogenase subunit HndC | DEG10120376 | NADH dehydrogenase I, F subunit | energy metabolism |
| END23_01852 | putative iron-sulfur-binding oxidoreductase FadF | DEG10350439 | hypothetical protein | - |
| END23_02093 | Bacterial protein of unknown function (YtfJ_HI0045) | DEG10050022 | hypothetical protein | - |
| END23_02311 | Transcriptional repressor PaaX | DEG10180239 | Phenylacetic acid degradation operon negative regulatory protein paaX | - |
| END23_02313 | Putative 1,2-phenylacetyl-CoA epoxidase, subunit D | DEG10340085 | hypothetical protein | - |
| END23_02314 | 1,2-phenylacetyl-CoA epoxidase, subunit C | DEG10180238 | Phenylacetic acid degradation protein paaC | - |
| END23_02318 | Acyl-coenzyme A thioesterase PaaI | DEG10070220 | Conserved hypothetical protein | Function unknown |
| END23_02331 | Transcriptional regulator DauR | DEG10050185 | hypothetical protein | - |
| END23_02358 | OsmC-like protein | DEG10150226 | hypothetical protein | Adaptation, protection |
| END23_02877 | Thymidylate kinase | DEG10500026 | hypothetical protein | - |
| END23_02886 | 2-succinyl-6-hydroxy-2,4-cyclohexadiene-1-carboxylate synthase | DEG10060267 | hydrolase, alpha/beta fold family | Unknown function |
| END23_03052 | hypothetical protein | DEG10480145 | polysaccharide biosynthesis protein | - |
| END23_03130 | hypothetical protein | DEG10410520 | membrane protein | - |
| END23_03234 | Collagen triple helix repeat (20 copies) | DEG10470208 | hypothetical protein | - |
| END23_03681 | hypothetical protein | DEG10200457 | hypothetical protein | - |
| END23_03971 | Phosphate-import permease protein PhnE | DEG10470310 | phosphonate ABC transporter, permease protein PhnE | - |
