# Supplementary material for: Comparative Genomics Reveals Metabolic Specificity of Endozoicomonas Isolated from a Marine Sponge and the Genomic Repertoire for Host-Bacteria Symbioses
Source: Microorganisms. 2019 Nov 30;7(12):635. doi: 10.3390/microorganisms7120635 (PMC6955870; doi:10.3390/microorganisms7120635)
Supplement: Supplementary file 1 [file microorganisms-07-00635-s001.zip › supplementaryMaterials/TableS4.docx]

**Supplementary Table S4.** List of eukaryotic-like proteins predicted in the genome of *Endozoicomonas* sp. OPT23

| **Locus ID** | **Product** | **InterPro ID** |
| --- | --- | --- |
|  | ORFs containing TPR domains |  |
| END23_00218 | Tetratricopeptide repeat | IPR019734 |
| END23_00362 | Tetratricopeptide-like helical domain superfamily | IPR011990 |
| END23_00443 | Tetratricopeptide-like helical domain superfamily | IPR011990 |
| END23_00802 | Tetratricopeptide-like helical domain superfamily | IPR011990 |
| END23_00828 | Tetratricopeptide-like helical domain superfamily | IPR011990 |
| END23_00851 | Tetratricopeptide-like helical domain superfamily | IPR011990 |
| END23_00919 | Tetratricopeptide-like helical domain superfamily | IPR011990 |
| END23_01093 | Tetratricopeptide repeat | IPR019734 |
| END23_01267 | Tetratricopeptide-like helical domain superfamily | IPR011990 |
| END23_01437 | Tetratricopeptide-like helical domain superfamily | IPR011990 |
| END23_01606 | Tetratricopeptide-like helical domain superfamily | IPR011990 |
| END23_01741 | Tetratricopeptide-like helical domain superfamily | IPR011990 |
| END23_03214 | Tetratricopeptide-like helical domain superfamily | IPR011990 |
| END23_03736 | Tetratricopeptide-like helical domain superfamily | IPR011990 |
| END23_03773 | Tetratricopeptide-like helical domain superfamily | IPR011990 |
| END23_03822 | Tetratricopeptide-like helical domain superfamily | IPR011990 |
| END23_03834 | Tetratricopeptide-like helical domain superfamily | IPR011990 |
| END23_03837 | Tetratricopeptide repeat | IPR019734 |
| END23_04033 | Tetratricopeptide repeat 1 | IPR001440 |
| END23_04082 | Tetratricopeptide-like helical domain superfamily | IPR011990 |
| END23_04089 | Tetratricopeptide-like helical domain superfamily | IPR011990 |
| END23_04096 | Tetratricopeptide-like helical domain superfamily | IPR011990 |
| END23_04116 | Tetratricopeptide-like helical domain superfamily | IPR011990 |
| END23_04153 | Tetratricopeptide-like helical domain superfamily | IPR011990 |
|  | ORFs containing Sel1 repeats |  |
| END23_01323 | Sel1-like repeat | IPR006597 |
| END23_03128 | Sel1-like repeat | IPR006597 |
| END23_03254 | Sel1-like repeat | IPR006597 |
| END23_03576 | Sel1-like repeat | IPR006597 |
|  | ORFs containing ANKs |  |
| END23_00128 | Ankyrin repeat-containing domain | IPR020683 |
| END23_01047 | Ankyrin repeat-containing domain | IPR020683 |
| END23_01129 | Ankyrin repeat-containing domain | IPR020683 |
| END23_01601 | Ankyrin repeat-containing domain | IPR020683 |
| END23_01801 | Ankyrin repeat-containing domain | IPR020683 |
| END23_04052 | Ankyrin repeat-containing domain | IPR020683 |
