# Supplementary material for: Comparative Genomics Reveals Metabolic Specificity of Endozoicomonas Isolated from a Marine Sponge and the Genomic Repertoire for Host-Bacteria Symbioses
Source: Microorganisms. 2019 Nov 30;7(12):635. doi: 10.3390/microorganisms7120635 (PMC6955870; doi:10.3390/microorganisms7120635)
Supplement: Supplementary file 1 [file microorganisms-07-00635-s001.zip › supplementaryMaterials/TableS5.docx]

**Supplementary Table S5.** List of predicted type VI secretion system effectors (T6Es) and type III secretion system effectors (T3Es) in the genome of *Endozoicomonas* sp. OPT23

| **Locus tag** | **PRODIGAL annotation** | **SecretEPDB annotation** |
| --- | --- | --- |
| **Predicted T6Es** | | |
| END23_00077 | Serine/threonine-protein kinase PknB | serine/threonine-protein kinase |
| END23_00120 | Pentapeptide repeat protein | Type III effector HopI1 |
| END23_00408 | Chaperone protein DnaJ | Type III effector HopI1 |
| END23_00781 | Type IV pilus biogenesis and competence protein PilQ | Outer membrane secretion protein Q |
| END23_00782 | Shikimate kinase 1 | Shikimate kinase |
| END23_00802 | Chaperone protein SicA | Low Calcium Response Protein H |
| END23_00803 | Secretion system effector C (SseC) like family protein | AopB |
| END23_00804 | hypothetical protein | Uncharacterized protein |
| END23_00810 | Yop proteins translocation protein L | Translocation protein L |
| END23_00876 | Type II secretion system protein D | Outer membrane secretion protein Q |
| END23_00967 | Shikimate kinase | Shikimate kinase |
| END23_00975 | Serine/threonine-protein kinase pkn5 | putative serine/threonine-protein kinase (TTSS effector protein) |
| END23_01039 | V-type ATP synthase alpha chain | Probable ATP synthase YscN |
| END23_01045 | V-type sodium ATPase subunit B | Probable ATP synthase YscN |
| END23_01146 | Protein-tyrosine phosphatase | Yop effector YopH |
| END23_01404 | Serine/threonine-protein kinase PrkC | serine/threonine-protein kinase |
| END23_01437 | Chaperone protein IpgC | Low Calcium Response Protein H |
| END23_01450 | Serine/threonine-protein kinase PrkC | putative serine/threonine-protein kinase (TTSS effector protein) |
| END23_01602 | Serine/threonine-protein kinase pkn5 | serine/threonine-protein kinase |
| END23_01699 | Tyrosine-protein phosphatase YopH | AopH |
| END23_01928 | Serine/threonine-protein kinase PknG | serine/threonine-protein kinase |
| END23_01966 | Transcription termination factor Rho | Probable ATP synthase YscN |
| END23_02089 | Secreted effector protein SseJ | Secreted effector protein SseJ |
| END23_02099 | hypothetical protein | Effector protein BopA |
| END23_02100 | hypothetical protein | Virulence protein IcsB |
| END23_02108 | ATP synthase subunit alpha | Probable ATP synthase YscN |
| END23_02110 | ATP synthase subunit beta | Probable ATP synthase YscN |
| END23_02112 | Bifunctional hemolysin/adenylate cyclase | Adenylate cyclase |
| END23_02113 | Serine/threonine-protein kinase StkP | putative serine/threonine-protein kinase (TTSS effector protein) |
| END23_02148 | HopJ type III effector protein | Type III effector HopJ1 |
| END23_02275 | Membrane-bound lytic murein transglycosylase B | Type III helper protein HopAJ1 |
| END23_02321 | Bifunctional protein PaaZ | Nodulation protein N |
| END23_02330 | putative peptidase | Methionine aminopeptidase |
| END23_02460 | Inositol phosphate phosphatase IpgD | Inositol phosphate phosphatase IpgD |
| END23_02484 | putative ATP synthase YscN | ATP synthase YscN |
| END23_02487 | Yop proteins translocation protein Q | Yop proteins translocation protein Q |
| END23_02488 | Surface presentation of antigens protein SpaP | Harpin secretion protein HrpW |
| END23_02490 | Surface presentation of antigens protein SpaR | HrpC protein |
| END23_02491 | Yop proteins translocation protein U | YscU (plasmid) |
| END23_02506 | Superoxide dismutase [Fe] | Superoxide dismutase |
| END23_02554 | putative enoyl-CoA hydratase 1 | Nodulation protein N |
| END23_02584 | hypothetical protein | Effector protein BopA |
| END23_02645 | Flagellum-specific ATP synthase | Probable ATP synthase YscN |
| END23_02647 | Flagellar assembly protein FliH | Translocation protein L |
| END23_02654 | Flagellar motor switch protein FliN | Yop proteins translocation protein Q |
| END23_02656 | Flagellar biosynthetic protein FliP | Harpin secretion protein HrpW |
| END23_02658 | Bacterial export proteins, family 1 | HrpC protein |
| END23_02659 | Flagellar biosynthetic protein FlhB | YscU (plasmid) |
| END23_02672 | putative enoyl-CoA hydratase 1 | Nodulation protein N |
| END23_02807 | Serine/threonine-protein kinase PrkC | putative serine/threonine-protein kinase (TTSS effector protein) |
| END23_02808 | Serine/threonine-protein kinase PknJ | putative serine/threonine-protein kinase (TTSS effector protein) |
| END23_02844 | Glycerophosphodiester phosphodiesterase, cytoplasmic | Avirulence protein AvrBs2 |
| END23_02857 | Calmodulin-sensitive adenylate cyclase | Adenylate cyclase |
| END23_03067 | Glycerophosphodiester phosphodiesterase, cytoplasmic | Avirulence protein AvrBs2 |
| END23_03120 | Leukotoxin | Endo-1,3-1,4-beta-glycanase ExsH |
| END23_03122 | Type II secretion system protein D | Outer membrane secretion protein Q |
| END23_03376 | Type II secretion system protein D | Outer membrane secretion protein Q |
| END23_03507 | Calmodulin-sensitive adenylate cyclase | Adenylate cyclase |
| END23_03658 | Transcription termination factor Rho | Probable ATP synthase YscN |
| END23_03718 | Methionine aminopeptidase | Methionine aminopeptidase |
| END23_03791 | Oxygen sensor histidine kinase NreB | MULTISPECIES: histidine kinase |
| END23_03860 | Pentapeptide repeat protein | Secreted effector protein PipB2 |
| END23_03941 | Serine/threonine-protein kinase PrkC | putative serine/threonine-protein kinase (TTSS effector protein) |
| END23_03970 | Serine/threonine-protein kinase PK-1 | serine/threonine-protein kinase |
| END23_04106 | Serine/threonine-protein kinase PknG | serine/threonine-protein kinase |
| END23_04116 | type III secretion low calcium response chaperone LcrH/SycD | Low Calcium Response Protein H |
| END23_04151 | Polyphenol oxidase | Polyphenol oxidase |
| END23_04213 | Serine/threonine-protein kinase PknB | serine/threonine-protein kinase |
| END23_04260 | Serine/threonine-protein kinase PrkC | Protein kinase YopO |
| END23_04282 | Bifunctional hemolysin/adenylate cyclase | Adenylate cyclase |
| **Predicted T6Es** | | |
| END23_00136 | Autoinducer 2-binding protein LsrB | RbsB |
| END23_00293 | ABC transporter periplasmic-binding protein YtfQ | RbsB |
| END23_01325 | Type VI secretion system effector, Hcp | Hypothetical protein Atu4345 |
| END23_01333 | Actin cross-linking toxin VgrG1 | Hypothetical protein PA2685 |
| END23_01335 | PAAR motif protein | - |
| END23_01467 | Periplasmic protein TorT | RbsB |
| END23_02443 | Type VI secretion system effector, Hcp | Hypothetical protein Atu4345 |
| END23_02451 | Actin cross-linking toxin VgrG1 | type VI secretion protein VgrG3 |
| END23_02739 | Lipase (class 3) | lipase |
| END23_02752 | D-galactose-binding periplasmic protein | RbsB |
| END23_03888 | catabolite repressor/activator | RbsB |
| END23_03916 | D-allose-binding periplasmic protein | RbsB |
